# Comparative metabolomic study of fungal foliar endophytes and their long-lived host *Astrocaryum sciophilum*: a model for exploring the chemodiversity of host-microbe interactions

**DOI:** 10.3389/fpls.2023.1278745

**Published:** 2023-12-19

**Authors:** Leonie Pellissier, Arnaud Gaudry, Salomé Vilette, Nicole Lecoultre, Adriano Rutz, Pierre-Marie Allard, Laurence Marcourt, Emerson Ferreira Queiroz, Jérôme Chave, Véronique Eparvier, Didier Stien, Katia Gindro, Jean-Luc Wolfender

**Affiliations:** ^1^ School of Pharmaceutical Sciences, University of Geneva, Centre Médical Universitaire (CMU), Geneva, Switzerland; ^2^ Institute of Pharmaceutical Sciences of Western Switzerland, University of Geneva, Centre Médical Universitaire (CMU), Geneva, Switzerland; ^3^ Mycology Group, Research Department Plant Protection, Agroscope, Nyon, Switzerland; ^4^ Department of Biology, University of Fribourg, Fribourg, Switzerland; ^5^ Laboratoire Evolution et diversité Biologique (Unité Mixte de Recherche (UMR) 5174), Centre National de la Recherche Scientifique (CNRS), Université Toulouse III (UT3), Institut de Recherche pour le Développement (IRD), Université Toulouse 3, Toulouse, France; ^6^ Université Paris-Saclay, Centre National de la Recherche Scientifique (CNRS), Institut de Chimie des Substances Naturelles, Gif-sur-Yvette, France; ^7^ Sorbonne Université, Centre National de la Recherche Scientifique (CNRS), Laboratoire de Biodiversité et Biotechnologie Microbiennes, Laboratoire de Biodiversité et Biotechnologies Microbiennes (LBBM), Observatoire Océanologique, Banyuls-Sur-Mer, France

**Keywords:** endophyte, fungi, metabolomics, molecular network, metabolite annotation, plant-fungi interactions, *Astrocaryum sciophilum*

## Abstract

**Introduction:**

In contrast to the dynamics observed in plant/pathogen interactions, endophytic fungi have the capacity to establish enduring associations within their hosts, leading to the development of a mutually beneficial relationship that relies on specialized chemical interactions. Research indicates that the presence of endophytic fungi has the ability to significantly modify the chemical makeup of the host organism. Our hypothesis proposes the existence of a reciprocal exchange of chemical signals between plants and fungi, facilitated by specialized chemical processes that could potentially manifest within the tissues of the host. This research aimed to precisely quantify the portion of the cumulative fungal endophytic community's metabolome detectable within host leaves, and tentatively evaluate its relevance to the host-endophyte interplay. The understory palm Astrocaryum sciophilum (Miq.) Pulle was used as a interesting host plant because of its notable resilience and prolonged life cycle, in a tropical ecosystem.

**Method:**

Using advanced metabolome characterization, including UHPLC-HRMS/MS and molecular networking, the study explored enriched metabolomes of both host leaves and 15 endophytic fungi. The intention was to capture a metabolomic "snapshot" of both host and endophytic community, to achieve a thorough and detailed analysis.

**Results and discussion:**

This approach yielded an extended MS-based molecular network, integrating diverse metadata for identifying host- and endophyte-derived metabolites. The exploration of such data (>24000 features in positive ionization mode) enabled effective metabolome comparison, yielding insights into cultivable endophyte chemodiversity and occurrence of common metabolites between the holobiont and its fungal communities. Surprisingly, a minor subset of features overlapped between host leaf and fungal samples despite significant plant metabolome enrichment. This indicated that fungal metabolic signatures produced in vitro remain sparingly detectable in the leaf. Several classes of primary metabolites were possibly shared. Specific fungal metabolites and/or compounds of their chemical classes were only occasionally discernible in the leaf, highlighting endophytes partial contribution to the overall holobiont metabolome. To our knowledge, the metabolomic study of a plant host and its microbiome has rarely been performed in such a comprehensive manner. The general analytical strategy proposed in this paper seems well-adapted for any study in the field of microbial- or microbiome-related MS and can be applied to most host-microbe interactions.

## Introduction

It is now established that plants are not strictly distinct entities but have evolved in association with highly complex and diverse microbial assemblages (Bonneville et al., 2020). Plant microbial symbiont communities (microbiota) and their collective genetic material constitute the plant microbiome (Turner et al., 2013). The plant microbiome is thought to play a key role in plant ecology and physiology, including plant fitness (growth and survival) (Dastogeer et al., 2020). It appears indeed that members of the microbiota play a role in regulating the immune system of the host plant by producing a wide range of metabolites that serve as signals involved in defense and competition, as well as in interactions and communication with the host plant (Brader et al., 2014; Vorholt et al., 2017). In return, the host plant also produces specialized metabolites that respond to symbiont colonization (Zhi-lin et al., 2007). Most signals are of a chemical nature and are mediated by lipids, peptides, polysaccharides, and volatile metabolites (Leach et al., 2017). Because of its ability to influence plant health and productivity and its potential impact on various applications (agriculture, drug discovery, etc.), the plant microbiome has received a great deal of attention in recent years within the scientific community. The plant that hosts a community is defined as an “holobiont”. This concept considers the multicellular host and its associated microbiota as a functional entity, in which evolutionary selection probably occurs between the host and microbes, as well as between microbes (Rosenberg and Zilber-Rosenberg, 2016).

In this study, we focused on endophytic fungi that reside within plant tissues. The evolution of symbioses is thought to be a key event leading to the territorialization of plants approximately 460 million years ago (Martin et al., 2017). Foliar fungal endophytes are ubiquitous and appear to influence the fitness of their hosts (U’Ren et al., 2019). In recent decades, they have been extensively studied for their ability to produce bioactive molecules as part of a defense response against pathogens for the host or for the fungi themselves (Christian et al., 2020). Unlike plant–pathogen interactions, endophytes can live a long time within their host, thus establishing a two-sided stable relationship involving interaction through specialized chemistry (Ludwig-Müller, 2015). Studies suggest that the host chemical profile is altered by the presence of endophytes and that host chemistry influences the composition and specificity of endophytic communities (Arnold et al., 2003).

Endophytes can influence host metabolism and induce the biosynthesis of specialized metabolites in the host. Some fungal endophytes have been documented to induce jasmonic acid and ethylene systemic defense responses in plants (Van Wees et al., 2008). It is well documented that endophytes and hosts can share parts of a particular metabolic pathway and produce a set of shared metabolites from similar precursors (Ludwig-Müller, 2015). The potential of endophytes to produce specialized metabolites that are biosynthesized by their host plants has been a major topic of interest in recent years. Examples include the microbial production of compounds with anticancer activities, such as paclitaxel and other taxanes (Stierle et al., 1993; Zhou et al., 2010), by a wide variety of endophytic species. One possible explanation for the ability of endophytes to synthesize plant-associated metabolites is that homologous gene clusters present in microbes and plants could be cross-activated by metabolites produced by stress, either from the host plant or endophytes (Howitz and Sinclair, 2008), and genetic recombination, such as horizontal gene transfer, allows endophytes to obtain genes encoding host biosynthetic enzymes (Tan and Zou, 2001; Tiwari and Bae, 2020). Endophytes can also metabolize host products (Glenn et al., 2001; Estrada et al., 2013; Ludwig-Müller, 2015). It seems clear that the specialized chemistry of the host is shaped by both that produced by the host and that from the endophytes, potentially creating a protective heterogeneous chemical composition in the plant (Vega and Blackwell, 2005). Conversely, the host can influence endophyte metabolism and shape the microbiome composition (Hansson et al., 2014). Plants can, for example mimic fungal pheromone oxylipins to regulate the development of fungi, production of mycotoxins, or attraction to insect pollinators (Gao and Kolomiets, 2009).

Given the involvement of chemistry in both the contribution of endophytes to host plant health and phenotype, and in the shaping of microbial communities, it is particularly relevant to conduct in-depth research on the chemical links between plants and endophytes (Vorholt et al., 2017). This would assist in the discovery and production of natural products of interest and unravel the potential of host–endophyte interactions. One of the main challenges is to understand the origin of a given metabolite and to differentiate whether it comes from the plant or its inhabitants, from both, or from the interaction between the two (Castro-Moretti et al., 2020).

In the context of plant microbiota, many studies have been conducted on endophytes to understand their ecological roles and ability to produce specialized metabolites (Lucaciu et al., 2019). The exploration of molecular and signaling mechanisms involved in plant-endophyte interactions has begun more recently with the use of model plants such as *Arabidopsis thaliana* (Kusari et al., 2013). Few studies have targeted a host and its community to evaluate whether the chemical production of the endophyte differs when it is isolated or in the host plant and to what extent endophyte-derived compounds contribute to the overall specialized chemical profile of the host. Despite recent research progress, notably in omics approaches, the chemical mechanisms involved in plant–endophyte interplay remain unclear, and data are still limited (Wani et al., 2015). This may be partly because those relationships are very complex; studying *in planta* phenomena from *in vitro* living conditions is limited, and high-quality chemical reference resources are lacking.

The recent emergence of high-throughput sequencing and meta-omics strategies, such as metagenomics and metatranscriptomics, has significantly improved the exploration of genes, transcripts, or proteins from millions of microbes and has also made it possible to analyze biochemical functions and interactions of the microbiome with the host (Subudhi et al., 2019). In particular, next-generation high-throughput sequencing techniques help to collect data on the genetic composition of microbial communities from diverse hosts and their relative phylogenetic groupings, enabling the determination of plant–microbe associations at a large community level (Epp et al., 2012; Donald et al., 2020). Metabolomic analysis provides information on the metabolites, sometimes numbering thousands of compounds, and can provide a “snapshot” of metabolite production (Castro-Moretti et al., 2020).

Untargeted UHPLC-MS/MS has become one of the methods of choice for this type of analysis because it allows the detection of a wide range of metabolites with high sensitivity while providing structural insight. With the latest advances, an increasing number of unique molecules can be detected, including signaling molecules (Van Dam and Bouwmeester, 2016), facilitating the detection and annotation of a larger number of metabolites secreted by plants and their associated microbes (Draper et al., 2011). Recent innovations in analytical chemistry and bioinformatics have addressed the challenge of annotating and studying relevant compounds from the huge datasets produced by these techniques, including the annotation of unknown compounds (Allard et al., 2016). Approaches such as molecular networking (MN) allow untargeted MS/MS data to be organized based on their spectral similarity, and thus should allow analytes to be grouped by chemical similarity. In particular, it compares metabolome composition of a wide range of samples, even for those in which few metabolites can be identified (Wang et al., 2016).

In this study, we aim to use these techniques to take a metabolomic snapshot of a leaf and its fungal community and study this plant-fungal interaction from a molecular point of view. We used *Astrocaryum sciophilum* (Miq.) Pulle as a host plant model (Kahn, 2008). This understory palm is endemic to the northeast region of the Amazon and is known for its remarkably long life cycle and resistance (Charles-Dominique et al., 2003). Its maturation age of approximately 170 years and leaves of up to 20 years old suggest that this palm can maintain a stable association with microbial communities over a substantial period. This implies that potential endophytes can survive and resist the plant environment, likely by developing interesting chemical strategies (Arnold et al., 2003). We hypothesized that reciprocal chemical interactions occur between plants and endophytic fungi via specialized metabolites that are potentially expressed in host tissues.

We propose an exploratory strategy using UHPLC-HRMS/MS profiling combined with molecular networking (MN) and state-of-the-art annotation techniques to investigate the metabolome of the host leaf, in parallel with the cumulative metabolome of its cultivable fungal endophytes. Our goal was to understand the proportion and origin of common compounds and to illustrate how both the host and endophyte communities contribute to the richness of holobiont metabolism.

## Results

### Experimental design

Our study aimed to comprehensively explore the metabolome of *A. sciophilum* leaf (host) and its endophytic fungal community, with the objective of conducting a thorough comparison. The primary goal was to identify common characteristics and potential chemical traits within the community that can be detected in the host.

Throughout this study, as mentioned in the introduction, it must be considered that, since *A. sciophilum* is studied in its natural habitat, all the metabolomic analyses of the entire leaf correspond to what has been defined as the holobiont (host leaf and associated microbes). To ensure clarity in the presentation of our findings, we refer to the “host” to represent the plant leaf tissue and the “community” to represent the fungal strains isolated from the “host”.

**Figure 1:**
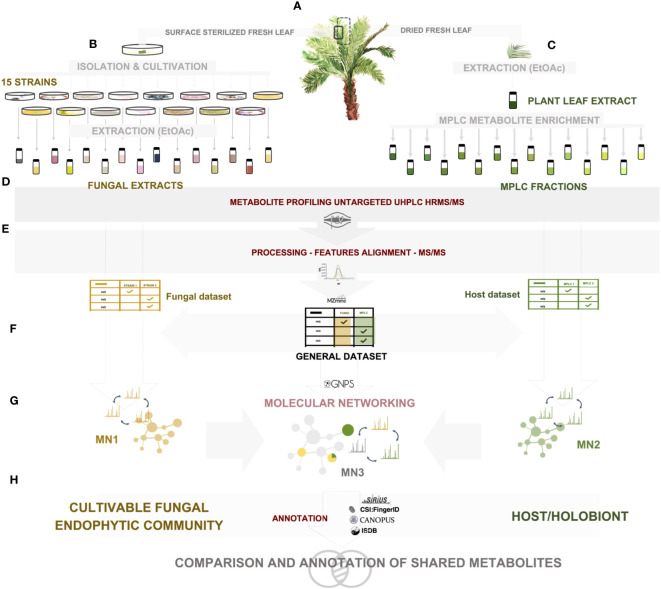
Experimental workflow of the study. **(A)** *A*. *sciophilum* leaf collection, **(B)** isolation of all cultivable fungal endophytic strains from sterilized fresh leaves, cultivation and extraction, **(C)** extraction of dried leaves and preparative MPLC fractionation for metabolite enrichment, **(D)** untargeted data-dependent UHPLC-HRMS/MS metabolite profiling, **(E)** HRMS-MS/MS data processing, **(F)** generation of two feature tables corresponding to fungal and host datasets, and creation of a common general dataset, **(G)** molecular network generation for each dataset: fungal MN1, Host MN2, general MN3, **(H)** annotation of each dataset through a combination of spectral matching against experimental and *in silico* databases, as well as computational and taxonomically informed methods.

The experiments were designed to explore the metabolomic data on both the fungal community and the host side, and to establish links between all the detected mass spectrometric features. The full metabolome dataset was then investigated to determine the extent of overlap between both datasets, first at the level of all detected features, and then by considering the identity of the metabolites involved. The general workflow is illustrated in **Figure 1**. Each step is detailed in the *Materials and methods* section.

To obtain a dataset on the fungal community, 15 cultivable strains were isolated as leaf endophytes of *A. sciophilum* (**Figure 1A**). Each strain was individually cultured on solid media and extracted with ethyl acetate (EtOAc) to yield 30 enriched extracts (**Figure 1B**).

To obtain the dataset for the host plant, the leaves of *A. sciophilum* were dried and extracted under similar conditions. Since it was expected that possible endophyte metabolite levels would be low, a strategy was designed to fractionate the extract at the preparative level and analyze all enriched fractions. To this end, the crude EtOAc extract was fractionated in one step using MPLC, yielding 47 enriched fractions (**Figures 1A, C**).

To obtain a detailed view of the metabolomes of the fungal endophytic community and the host leaf (through the cumulative metabolite profiles of all fractions and the crude extract), all samples were profiled using data-dependent UHPLC-HRMS/MS (**Figure 1D**).

To ensure reproducibility, all samples were acquired in triplicate in a single series of analyses, and great care was taken to avoid any possible cross-interference. For general data, only the features detected in at least all replicates of a given sample were considered.

Uniform MZmine peak picking (Pluskal et al., 2010) on all 234 samples (30 fungal and 48 leaf samples in analytical triplicates) allowed the creation of a unique feature list (general dataset) of 24,096 features in the positive ionization mode (PI) and 10,792 in the negative ionization mode (NI) (**Figure 1E**). In the first instance, the feature list of all fungal samples on one side and the feature list of all host-leaf fractions and extract on the other were considered for data processing and organized into two molecular networks (MN1 and MN2) (**Figure 1F**). [fungal community metabolome (fungal dataset): 11,072 features in PI (5,982 in NI); host-leaf metabolome (host dataset): 13,884 features in PI (5,503 in NI)]. We focused mostly on PI data in this study because, at present, the chemical class and *in silico* algorithms available for MS/MS spectral prediction are mostly efficient for this ionization mode and, overall, most of the features were detected in PI. Mass spectrometry data were deposited in the MassIVE public repository (n° MSV000088516).

Both datasets were then used in a combined MN (MN3) representing the whole dataset to evaluate the number of metabolites shared and/or expressed in the fungal community and host (**Figure 1G**). All steps and tools used for MN creation are detailed in the *Materials and methods* section.

Most features showing overlap between the fungi (specific to single strains or shared by multiple strains) and the host were investigated in more depth for annotation (**Figure 1H**).

Metabolite annotation was first performed at the chemical class level using CANOPUS (Dührkop et al., 2021). This tool is independent of any database search and provides structural data at the chemical class level, even for unknown compounds. This approach permitted class annotation of all nodes within the general dataset and retained this information in both fungal and host datasets. Further compound-specific annotation was performed considering the taxonomic information and MN cluster consistency (see *Materials and methods*). Concomitant analysis of standards and previously isolated molecules validated some annotations that were propagated from features to features within a cluster.

A summary of the main features of the fungal dataset is provided in *Section 2*. Similarly, the major families of compounds in the host-leaf-enriched metabolome are discussed in *Section 3*. Finally, the annotated features common between the fungal and host datasets are discussed in detail in *Section 4* at the level of each individual feature.

### Metabolome data on all cultivable strains of the fungal community

To obtain a dataset on the fungal community, several fragments of fresh leaves of *A. sciophilum* were surface-sterilized, placed, and cultivated on individual Petri dishes. This enabled the isolation and identification of 15 individual strains that were cultured on solid media on a small scale (**Figure 1A**). The species identified for these strains are listed per strain in **Supplementary Table 1**. The isolation yielded one Mucoromycota and 14 Ascomycota, among which 11 were Sordariomycetes and three Dothideomycetes. The composition of this community reflected the taxonomic trends observed in foliar endophytic fungal communities. Indeed, Dothideomycetes and Sordariomycetes represent the majority of foliar endophyte species and account for 75% of the endophytes isolated worldwide (Arnold, 2007). The genera include those commonly isolated from tropical trees, as illustrated in (Roy and Banerjee, 2018), namely *Colletrotrichum* Corda*, Curvularia* Boedijn*, Pestialotiopsis* Steavaert, and *Fusarium* Link. This indicated that the sampled foliar endophytic fungal community was representative of what is generally encountered in natural ecosystems.

Individual strains were extracted with EtOAc to yield 30 enriched extracts and analyzed according to the generic metabolite profiling method detailed in the experimental design (see *Materials and methods*) (**Figures 1B, D**). The MN corresponding to the fungal dataset (MN1) for PI data permitted grouping of 11,072 spectra in 6,924 clusters (5,982 spectra in 2,755 clusters in NI).

**Figure 2:**
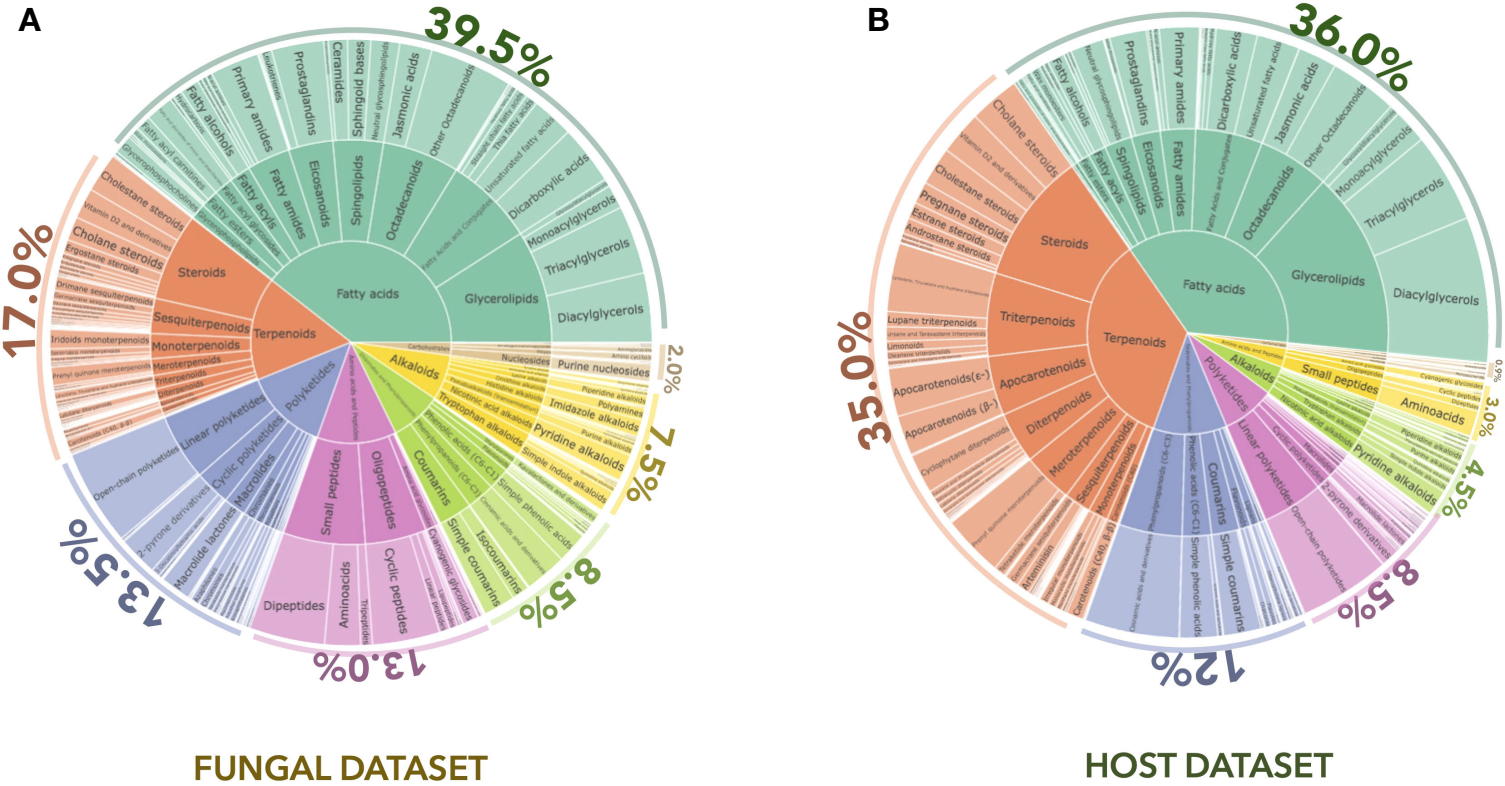
CANOPUS chemical annotation of the **(A)** fungal and **(B)** host datasets. Hierarchical sunburst diagrams representing the main chemical classes were established using the NPclassifier. The sunburst displays the pathway in the inner circle, the superclass in the middle circle, and the various chemical classes in the external circle. The number of associated features for each category was displayed based on the area size. The proportion of features annotated per chemical pathway was represented as a percentage. An interactive view of the plots is available at Interactiveplots^1^.

The first annotation step with CANOPUS enabled us to obtain a global overview of the compound classes measured in fungal samples. In **Figure 2A**, the chemical pathway repartition is color-mapped on the MN, highlighting the structural type for each node (**Supplementary Figure 1A**). The main chemical classes were represented using a hierarchical sunburst diagram. Each NP classifier category was assigned to a circle area, with their subcategory circles nested inside, while the number of associated features for each category is displayed via area size. The innermost circle was at the top of the hierarchy. A detailed and interactive view of sunburst diagrams is available at Interactiveplots^1^. Overall, CANOPUS enabled the chemical class annotation of 4,268 features out of 11,072, with the following percentage repartition at the pathway level: 39.5% fatty acids, 17.0% terpenoids, 13.5% polyketides, 13.0% amino acids and peptides, 8.5% shikimates and phenylpropanoids, 7.5% alkaloids, and 2.0% carbohydrates.

**Figure 3:**
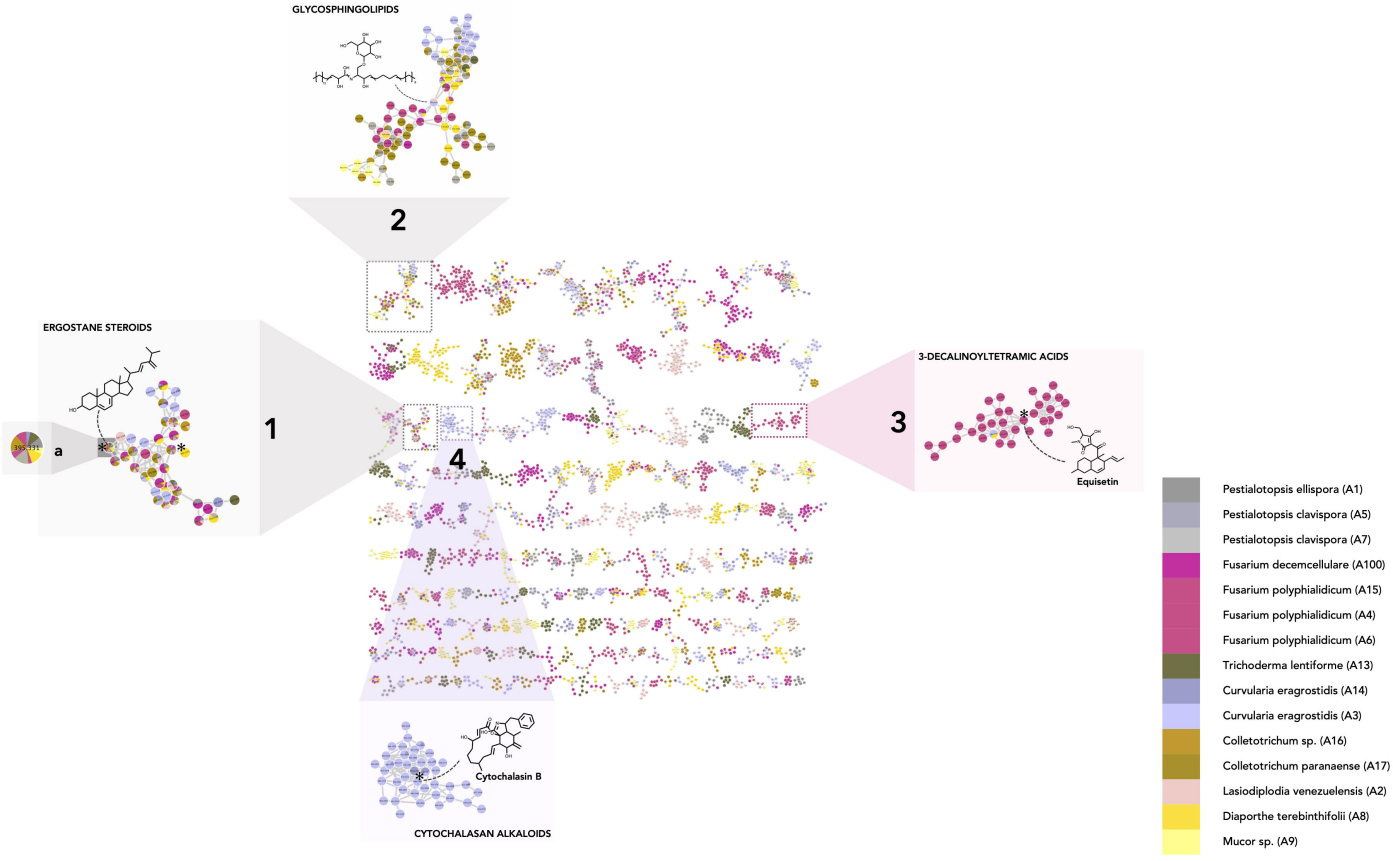
Molecular network of the fungal dataset (MN1). Nodes are colored based on their taxonomic attributes to denote their origin. Pie charts within each node indicate the distribution of MS intensities of each feature in the entire sample set. Nodes with single colors are characteristic of metabolites that occur in only one specific strain. Nodes with multiple colors denote shared metabolites. (1–2) Clusters shared by multiple species, highlighting either specific shared features and/or shared chemical classes. (1a) Node corresponding to a feature detected in multiple species and annotated as 24(28)-dehydroergosterol. (3–4) Clusters specific to particular strain(s) (single color nodes) (3: *Fusarium polyphialidicum*, 4: *Curvularia eragrostidis*).

The processing of all the information in the MN allowed us to highlight the features shared between two or more strains and to interpret these compositional overlaps. Annotation of features confirmed by a standard or isolated molecule is highlighted with a star (✻). Taxonomical information was also represented by colors on the MN (one color for one strain and shades of the same color for different strains of the same genus) to provide an overview of the strain of origin for each node (**Figure 3**).

To evaluate the major trends in the dataset, metabolite chemical classes shared between a few endophytic species were identified. In practice, to assess overlap rates, individual features detected in more than three samples from different species, *i.e.*, nodes tagged with three or more colors (one color corresponds to one species). were examined. For example, the feature corresponding to 24(28)-dehydroergosterol was shared by seven species and is highlighted in cluster **Figure 3**_1a Most of the features of the associated cluster were annotated as ergostane steroids by CANOPUS and were shared by multiple species (**Figure 3**_1).

Common classes of metabolites were also highlighted when specific features were not shared between samples but were linked through a shared cluster (nodes having one color only, grouped with nodes of another color). This indicates that they have common substructures, leading to spectral similarities and, thus, potentially common biosynthetic pathways. This is illustrated in cluster **Figure 3**_2, which is mostly annotated as glycosphingolipids. The other clusters displaying common fungal metabolite classes are shown in **Supplementary Figure 2**. These corresponded mostly to glycerolipids (**Supplementary Figure 2**_1) (Griffin, 1996), purine nucleosides (**Supplementary Figure 2**_2) (Moffatt and Ashihara, 2002), amino acid and peptide derivatives including cyclic peptides (**Supplementary Figure 2**_3) (Wang et al., 2017), dipeptides (cluster 17, **Supplementary Figures 2**_4, 5) (Mishra et al., 2017), polyketides (**Supplementary Figure 2**_6), and shikimates and phenylpropanoid derivatives (**Supplementary Figure 2**_7) (Tzin et al., 2012; Cox et al., 2018). Overall, these annotations make sense with respect to what is known for fungal metabolites (Zhang et al., 2006). The occurrence of such compounds in most species was expected, as ergostane steroids are sterols specifically found in fungal cell walls (Nes et al., 1989; Klemptner et al., 2014), and glycosphingolipids are ubiquitous components of the fungal membrane (Fernandes et al., 2018).

Clusters and features found to be specific to strains and/or particular species were also observed (clusters containing mostly single-color nodes). For instance, the cluster in **Figure 3** **_3** contains nodes coming principally from *Fusarium concolor* Reinking (taxon synonym *Fusarium polyphialidicum*) strains (A4, A6), and most of the nodes were annotated as compounds from the 3-decalinoyltetramic acid class (superclass: cyclic polyketides). Within this cluster, for example, the feature at *m/z* 374.232, t_R_ 4.83 (molecular formula C_22_H_31_NO_4_) was annotated as equisetin by SIRIUS and was fully identified by the injection of a standard. This is consistent with the fact that this compound and its derivatives have already been specifically isolated from *Fusarium* sp. (Sims et al., 2005). Other examples include a cluster specific to *Curvularia* sp. strains (A3 and A14) (**Figure 3**_4), which were annotated as cytochalasan alkaloid metabolites. Within this cluster, the feature at *m/z* 480.274, t_R_ 2.99 was annotated by SIRIUS as cytochalasin B and the annotation was confirmed by injection of a standard. This is consistent with previous reports indicating the presence of cytochalasin A or B in *Curvularia* sp. (Khiralla et al., 2019). Additional-specific clusters were identified and highlighted (**Supplementary Figures 2**_8–15): jasmonate derivatives (**Supplementary Figure 2**_8) and naphtalene polyketides (**Supplementary Figure 2**_15) in *Lasiodiplodia* sp. (Salvatore et al., 2020), shikimates, and phenylpropanoids (coumarins and isocoumarins) in *Diaporthe* sp. (Luo et al., 2018) (**Supplementary Figure 2**_9), azaphilones in *Trichoderma lentiforme* (Pang et al., 2020) (**Supplementary Figure 2**_10), histidine alkaloids in *Fusarium* sp. (Wen et al., 2015; Ma et al., 2022) (**Supplementary Figures 2**_11, 12). Overall, the annotation results were proved consistent with the literature data on the corresponding fungal species or genera, even before taxonomic re-ranking.

Altogether, analyses of the dataset revealed a consistent chemical class annotation for ubiquitous fungal compounds, as well as at a species-specific level. The metabolite profiles also revealed a good clustering trend based on the taxonomy of the fungi. PCoA (Bray–Curtis dissimilarity metrics) and Hierarchical Cluster Analysis (HCA, single linkage method) were performed based on the metabolite fingerprint (PI) of the 15 strains using the fungal-dataset feature table and showed that samples were clustered together according to their taxonomic affiliation (**Supplementary Figure 3**). This showed that the set was informative at the chemical level, thus enabling exploration of data on the fungal community.

### Deep metabolome data on the palm host leaf *Astrocaryum sciophilum* (holobiont)

The MN corresponding to the host dataset (MN2) (crude extract and enriched fractions) for PI data allowed the grouping of 13,884 spectra into 8,315 clusters.

Using CANOPUS, the initial annotation phase allowed us to obtain a comprehensive overview of the compound classes measured in the host leaf samples by benefiting from the enrichment procedure described in **Figures 1A, C** in *Experimental design*. All annotations were processed in the same way as for the fungal dataset, the chemical pathway repartition color-mapped on the MN, and the main chemical classes represented as a hierarchical sunburst diagram (**Figure 2B**, Interactiveplots^1^). Overall, CANOPUS enabled the chemical class annotation of 5,957 features out of 13,884, with the following percentage pathway repartition: 36.0% fatty acids, 35.0% terpenoids, 12.0% shikimates and phenylpropanoids, 8.5% polyketides, 4.5% alkaloids, 3.0% amino acids and peptides, and 0.9% carbohydrates (**Figure 2B**).

**Figure 4:**
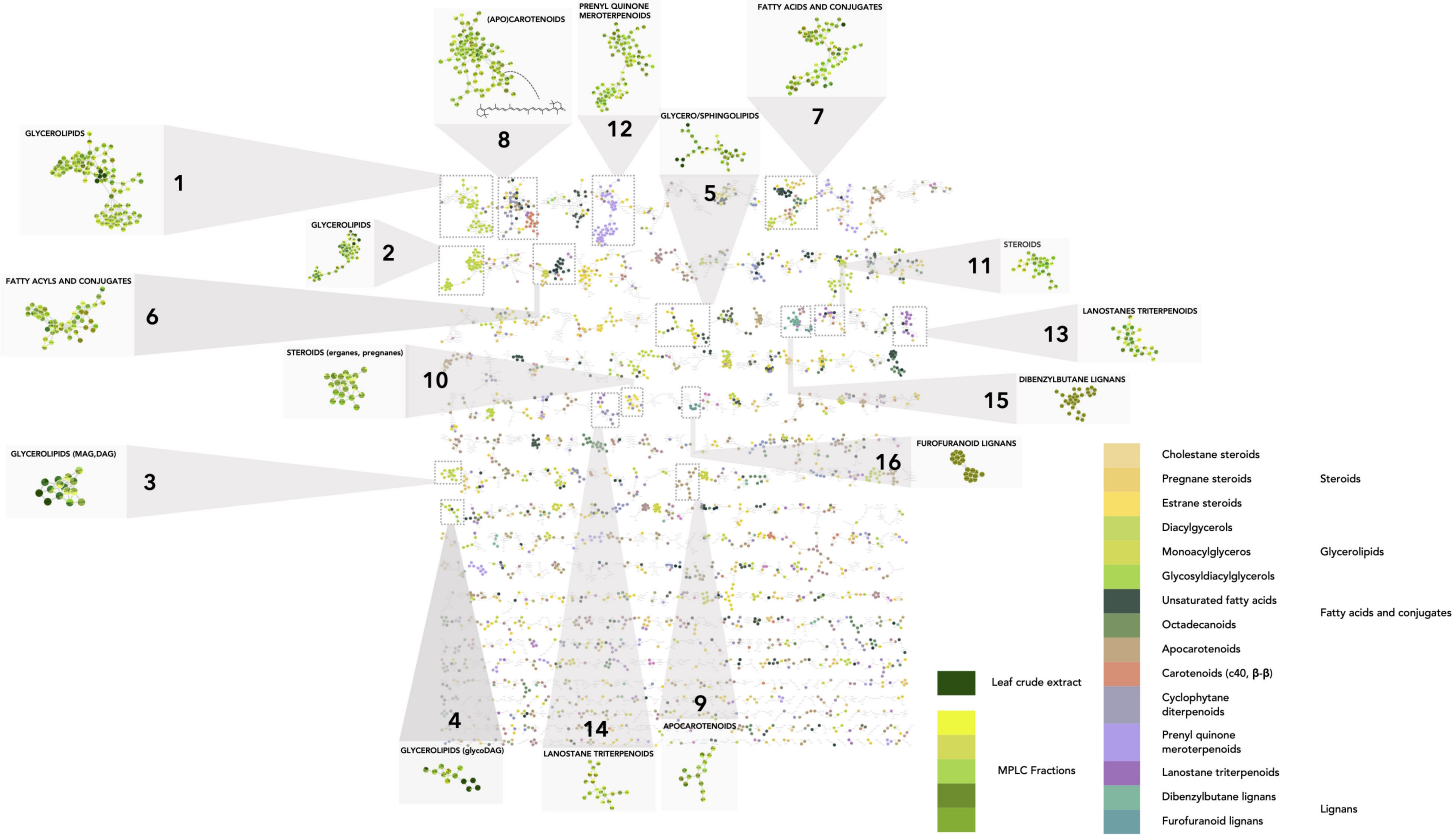
Molecular network on the host dataset (MN2). In the host MN, the nodes are colored based on their NPclassifier chemical class. In zoomed clusters, pie charts within each node indicate the distribution of MS intensities of each feature in the leaf crude extract and/or MPLC fractions. (1–16) Clusters of representative leaf metabolite classes.

The chemical CANOPUS class repartition was color-mapped onto the MN (**Supplementary Figure 1B**). The sample type (EtOAc extract or MPLC fraction) is also represented by colors (shades of green) on the MN to provide an overview of the sample(s) of origin for each node (zoomed squares, **Figure 4**). This enabled us to obtain a global overview of the compound classes measured in plant extracts and fractions. Well-represented chemical classes are highlighted: glycerolipids (**Figures 4**_1–4), sphingolipids (**Figure 4**_5), fatty acids and conjugates (**Figure 4**_6, 7), (apo)carotenoids (**Figure 4**_8, 9), steroids (**Figure 4**_10, 11), terpenoids (**Figure 4**_12), and triterpenoids (**Figure 4**_13, 14). Some chemical classes, such as glycerolipids and fatty acid conjugates, were also detected in the crude extract, indicating that they may be the main metabolites. Some were well spread in the fractions or were more specific to one fraction, such as lignans, which were detected mostly in fraction 33 (**Figure 4**_15–16). Some have been reported to be produced by members of the Arecaceae family, including lanostane triterpenoids reported in *Sabal* sp. (El-Dib et al., 2004), cholestane steroids (Tapondjou et al., 2015), flavonoids (de Oliveira et al., 2013) (**Supplementary Figures 4A–C**), and even by the genus *Astrocaryum* such as carotenoids (Noronha Matos et al., 2019) (**Supplementary Figure 5**). Within the carotenoids in cluster **Figure 4**_8, the feature at *m/z* 551.42, t_R_ 6.56, was annotated as β,β-caroten-4-one, and annotation was confirmed by the injection of a standard.

MN processing also allowed visualization of the detection status of each feature: only in the crude extract (**Supplementary Figure 6A**_1), only in the fractions (one or more) (**Supplementary Figure 6A**_2), or both (**Supplementary Figure 6A**_3). Features found only in the extract may be compounds degraded in the fractions or retained in the MPLC column. As shown in **Supplementary Figure 6B**, the sum of all the features detected in the fractions was much higher than that obtained by profiling only the raw extract. Indeed, the features contained in the crude EtOAc extract accounted for only 3.7% of the total features detected (510 features detected in the crude extract and 13,592 in the MPLC fractions). The enrichment process effectively revealed approximately 30 times more features than total crude extract profiling. These features were well distributed across the MPLC fractions. Hence, the enrichment step is important to improve the detection of minor compounds and shared features in the MN comparative analysis (**Supplementary Figure 7**).

### Global comparison of the entire metabolome dataset: host leaf (holobiont) and foliar fungal endophytic community

In the next phase, as both UHPLC MS/MS datasets belonged to the same data table and all sample origin metadata were available for each feature, the process described in *Sections 2 and 3* could be applied to the entire general dataset (fungal + host). This ensured a consistent connection between all the features and their corresponding annotations. Consequently, a single MN encompassing all filtered fungal and host features was created (MN3), highlighting the correlations existing among all detected feature spectra (see *Materials and methods*, MassIVE Dataset n° MSV000088516). This led to the grouping of 24,096 spectra into 14,473 clusters.

All features that were directly shared between the endophytes and the host are highlighted. Additionally, features associated with fungi were grouped together in clusters associated with plants, probably demonstrating links at the level of shared classes or structural skeletons. Conversely, this approach also revealed clusters or features that were detected only in host or fungal samples.

#### Comparative analysis of the general dataset

**Figure 5:**
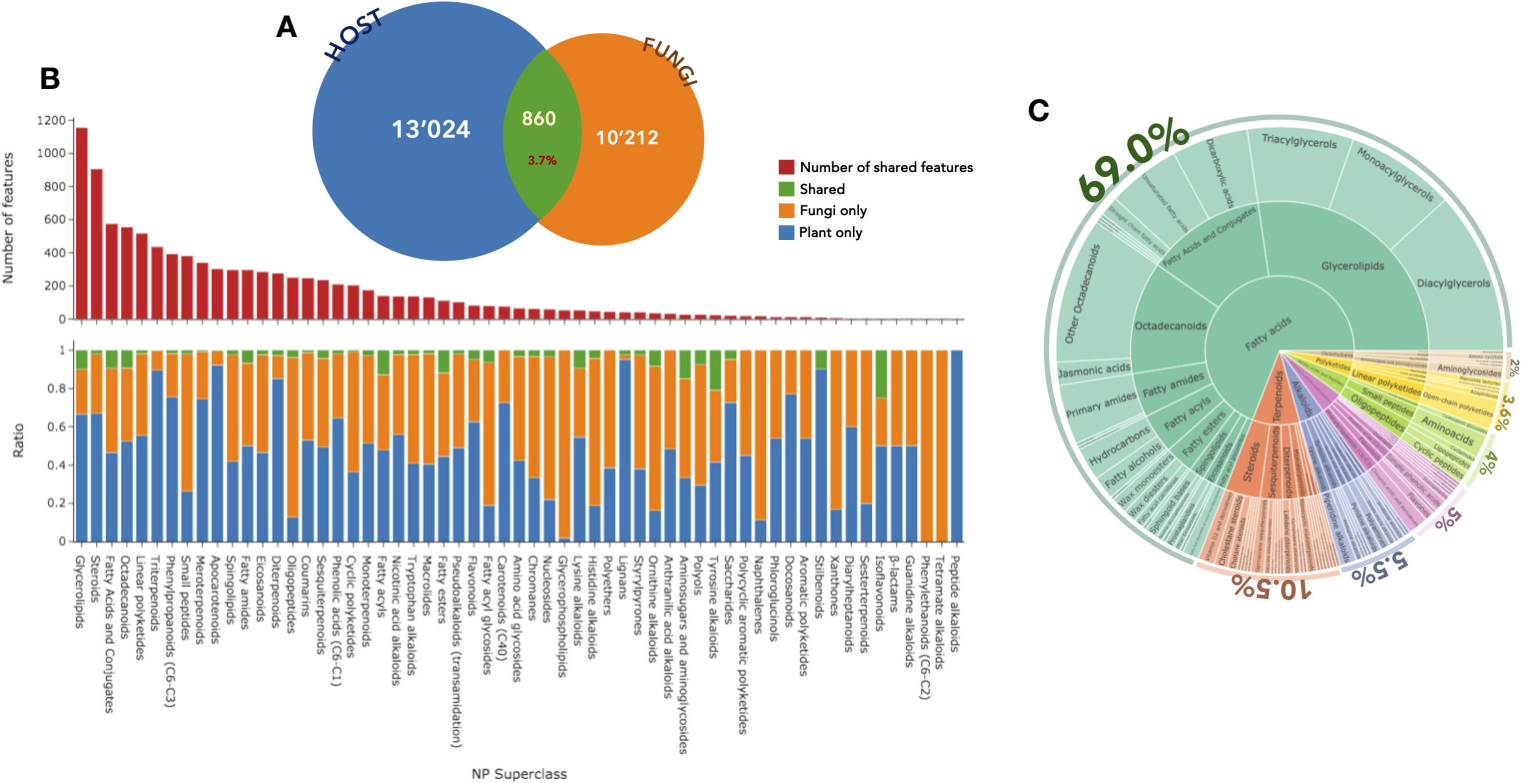
**(A)** Number of features detected in positive ion mode in the host (blue) and fungal (orange) datasets and the number of shared features (green). **(B)** Distribution and number (red) of these annotated features according to the NPclassifer superclasses in the fungal (orange), plant (blue), or shared (green) datasets. **(C)** Hierarchical sunburst diagram of shared features, organized in the same way as in **Figure 2**. An interactive view of the plots is available at Interactiveplots^1^.

The first survey of the data showed that, at the cluster level, 425 clusters were shared between the plant and the fungal community, potentially corresponding to compounds of a similar chemical class. At the feature level, among the 24,096 features detected in PI, approximately 860 (4%) were detected both in the plant host and in the fungal community (**Figure 5A**). In NI, 693 (6%) were shared among the 10,792 detected features.

If the same comparison was made only with the metabolite profiling obtained from the raw extract, this would indicate only 70 shared features (0.3%). Therefore, the significant host leaf extract enrichment process was effective in revealing approximately 10 times more shared features (**Supplementary Figure S8**).

Among the shared features, each endophytic strain revealed between 18% and 42% of shared features with the host (150 to 500 features per strain) (**Supplementary Figure S9**). Considering only the number of shared occurrences, it seemed that among all endophytes, some strains (A2, A7, A15, A100, and A14) had a metabolic signature detectable at the leaf level, while some (A8, A9, and A13) had a more subtle expression. Cinsidering the possible analytical and physiological variations, this suggests that some strains were more expressive than others. This aspect should be balanced by the fact that the shared metabolites correspond to fungal-specialized metabolites.

### Annotation of shared features (case studies)

To conduct a thorough study, the dataset needs to focus on annotated/identified metabolites present in both endophytes and the host leaf.

In addition to the annotation obtained with CANOPUS (see parts 2 and 3), a complementary annotation phase was used (ISDB with taxonomic and consensus chemical classification reweighting), focusing on the PI data.

Furthermore, the use of standards and previously isolated molecules enabled to unambiguous identification of more than 40 features and facilitated the overall annotation process through the propagation of more than 50 additional features (**Supplementary Figures S10**-**15**; **Supplementary Table S2**).

Among the 24,096 features from the general dataset, the CANOPUS chemical class led to the annotation of 20,314 features at the pathway level (**Supplementary Figure S16A**), 16,156 at the superclass level (**Figure 5B**), and 9,808 at the class level (**Supplementary Figure S16C**). In the histogram displayed in **Figure 5B**, the total number annotated features per chemical category is indicated at the top level along with the repartition of the features in the host leaf (blue), the fungal community (orange), or both (green). Considering only the features shared between the host and the fungi, the annotations were organized as a hierarchical sunburst diagram (**Figure 5C**, detailed and interactive view available at Interactiveplots^1^). The chemical pathway annotation of the shared features was as follows: 69.0% fatty acids, 10.5% terpenoids, 5.5% alkaloids, 5.0% shikimates and phenylpropanoids, 4.0% amino acids and peptides, 3.6% polyketides, and 2.0% carbohydrates.

Analysis of the general dataset based on the NPclassifier ontology (Kim et al., 2021) revealed that the chemical pathway most commonly found to be shared between plants and fungi was that of fatty acids. An overview of these data indicated that features related to terpenes and shikimates were mostly found in the host plant leaves, whereas amino acid and peptide derivatives were mostly detected in the fungal endophytic community. The fatty acids and polyketides spread well between the two. This trend was already reflected in the separate analysis of the host and fungal datasets (see **Figure 2**, sunbursts).

The frequency of the features at each superclass level is then considered. For this, all categories regrouping more than 15 features were analyzed, and specificity was assessed when more than 90% of the features were found either in the host or the fungi. The main specific superclasses of the host were triterpenoids, apocarotenoids, diterpenes, and lignans. The fungi contained oligopeptides, glycerophospholipids, and naphthalenes. Varying percentages of shared features and clusters were also observed.

To better document host–endophyte interactions, the most frequently shared features were initially analyzed category by category, considering what has been reported in the literature in general in plant–fungi interactions.

#### Shared features well spread between fungal and host datasets

The data indicated that most of the shared metabolites belonged to fatty acids, and the following superclasses: glycerolipids, fatty acids and conjugates, octadecanoids and steroids, and more specifically at the class level, to mono-, di-, and triacyclglycerols (MAG, DAG, TAG), dicarboxylic acids, unsaturated fatty acids, and octadecanoids (see Interactiveplots^1^). In the first assessment, these findings appeared logical, because these types of metabolites are known to be produced by both plants and fungi. Fatty acids are ubiquitous in nature and represent a major chemical class involved in physiologically important processes, such as membrane structure, energy storage, and signaling pathways (Athenaki et al., 2018; He et al., 2020).

Specific features or analogs of fatty acids commonly detected in one or more endophytic fungi and in the host leaf were studied. A partial feature table and annotations can be found in **Supplementary Table 2** and the full peak list is provided in **Supplementary Table 3**.

##### Shared glycerolipids

**Figure 6:**
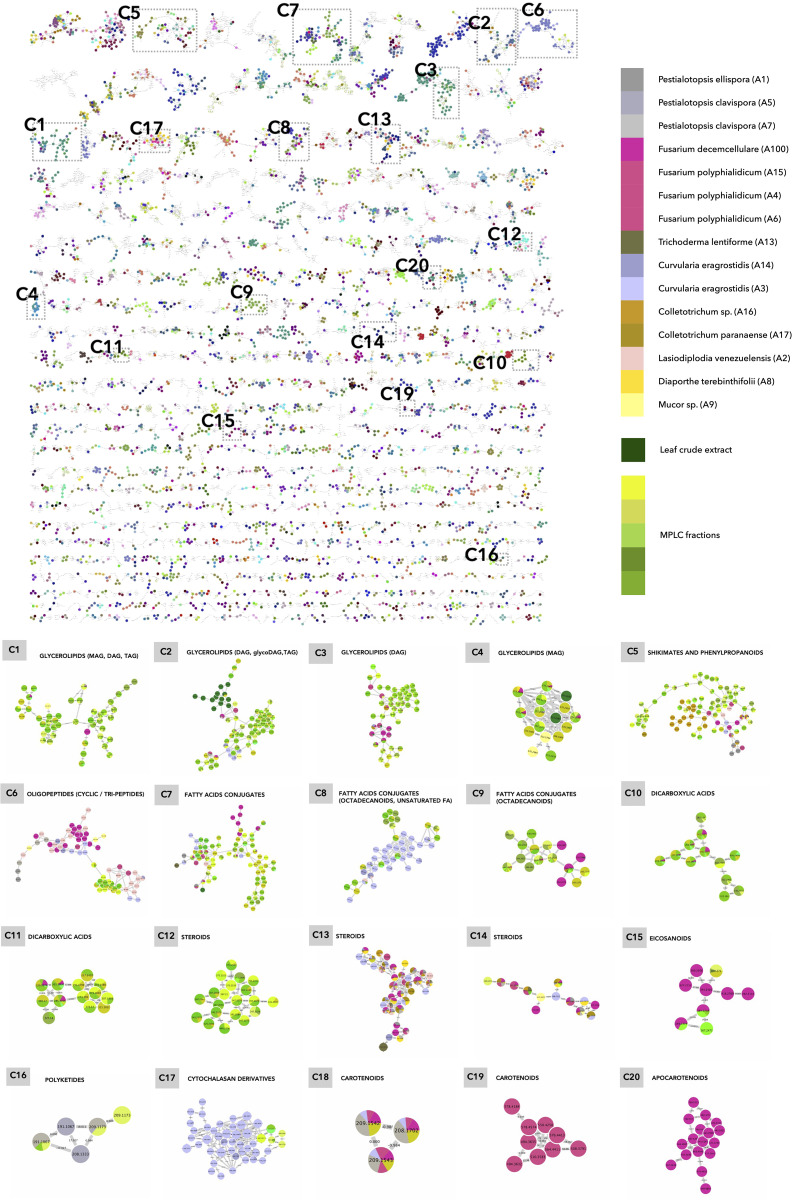
Molecular network of the general dataset (MN3). The nodes are colored based on their NPclassifier chemical classes. In zoomed clusters, pie charts within each node indicate the distribution of MS intensities of each feature in the leaf crude extract and/or in the MPLC fractions (green colors) and/or in one or more fungal strains. (C1–C20) Examples of clusters with shared features highlighted in MN3.

**Figure 7:**
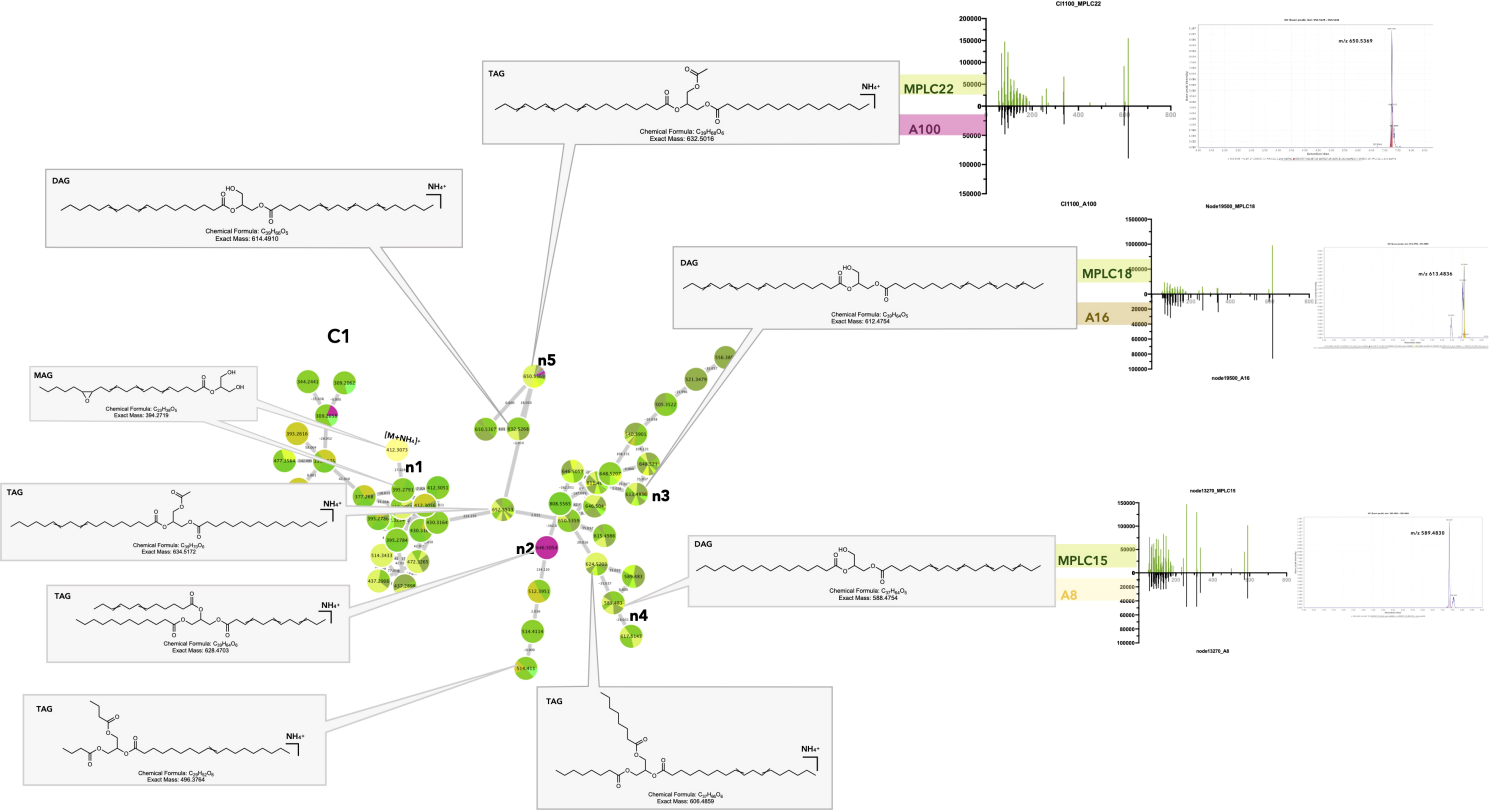
Selected annotations of cluster C1 from **Figure 6**, including various glycerolipids. Annotated features n3, n4, and n5 are shared between the host leaf and particular strains of *Colletotrichum* sp. A16, *Diaporthe terebinthifolii* A8, and *Fusarium decemcellulare* A100 (for the color code, see **Figure 6**). n2 corresponds to a specific glycerolipid of strain A100, detected in culture, but not in the leaf. Representative MS/MS spectra of some annotated glycerolipids and the single ion trace of the features across samples are displayed for shared features.

The distribution histogram shows that the most frequently shared features corresponded to glycerolipids (GL) (**Figures 5A, B**, Interactiveplots^1^, corresponding clusters **Figures 6**_C1–4). This is particularly true for triacylglycerols (TAGs) and diacylglycerols (DAGs). In C1, most of the features originated from the host samples, indicating that TAGs and DAGs were abundant in the leaves (**Figure 7**). A detailed analysis of these shared features showed that the feature n3 at *m/z* 613.483, t_R_ 7.11 (613.483@7.11) was found in the host as well as in *Colletotrichum* sp. (A16) (**Figure 7**_C1_n3). This feature corresponds to the protonated molecule [M + H]^+^ of a diacylglycerol (DAG) with a molecular formula of C_39_H_64_O_5_ (Δppm 1.5) and was annotated as DAG (18:3n3/18:3n3) (see **Supplementary Table 2**). The figure also shows the extracted ion chromatogram for the shared features in either the fungal strain or the MPLC fraction profile. This allowed us to verify that the alignment, particularly in terms of retention time, was correct for the features found to be shared. The MS/MS fragmentation patterns for the feature in the strain or plant were associated and compared, and it was ensured that the metabolites found to be shared by both samples had the same hypothetical structure (**Figure 7**_C1_n3). For example, cluster C4 showed that several isomers of MAG 2-monolinolenin were shared between some fungal strains and plant leaves (**Supplementary Figures 17**_C4_n1–n3_feature n1, 353.2696@5.1).

The results obtained for the various MAGs, DAGs, and TAGs detected were coherent because these metabolites are known to be produced by both plants and fungi. GLs are basic components of animals, plants, microbial cell membranes, and lipid body fractions (role in carbon storage for energy, lipid metabolism, and lipid-mediated signaling pathways in plants and fungi) (Zhang et al., 2012; Lastovetsky et al., 2016; Siebers et al., 2016; Qiu et al., 2020). GLs might also have a role in the response to microbial pathogenic or beneficial invasion, going from signal reception at the host plasma membrane to transduction and downstream defense pathways through induced or acquired systemic resistance (Siebers et al., 2016). In pathogenic interactions, DAGs can act as secondary messengers for the regulation of developmental processes but also modulate the lipid composition of the host membrane for colonization and invasive growth (Sadat et al., 2014). In symbiotic relationships, especially at the root level, it was demonstrated that DAGs and TAGs production was increased during arbuscular mycorrhizal formation, probably to create a larger exchange surface and energy storage, and to generate JA derivatives during defense responses. The fungal fatty acid palmitvaccenic acid stored as TAG is used as a marker of mycorrhizal colonization (Siebers et al., 2016; Macabuhay et al., 2021). MAGs, such as monolinolenins, could be involved in plant defense, as they have been shown to accumulate upon fungal infection and to interact with the membrane of diverse pathogenic bacteria or fungi (Kusumah et al., 2020; Toljamo et al., 2021). Overall, it is difficult to say with this dataset if the shared specific GLs detected in leaves result from symbiosis with the fungal strains for which we could clearly demonstrate these compounds when cultured *in vitro*, or if the leaf or root itself is producing them (Agostini-Costa, 2018).

##### Shared unsaturated fatty acids

Unsaturated fatty acids and octadecanoids were also frequently detected in both host and the fungi. These NP classifier categories are strongly linked and discussed together. Examples of the corresponding clusters are shown in the MN (**Figures 6**_C7–9).

In cluster C7, several features were classified as unsaturated fatty acids and the others as octadecanoids (**Supplementary Figures 18**_C7_ n1–n13). Several ions correspond to linoleic acid or its isomers. Some were found to be specific to fungal strains (*F. polyphialidicum* A4 and A6, *Lasiodiplodia venezuelensis* A2, *Curvularia eragrostridis* A3, *Neopestalotiopsis ellipsospora* A1) (**Supplementary Figures 18**_C7_n1–n3), and some were detected only in the plant and annotated as fatty acids found in Arecaceae (**Supplementary Figures 18**_C7_n4–n8) (de Oliveira et al., 2016; Agostini-Costa, 2018). Several of them were shared with the host: 279.2321@6.74, annotated as alpha-linoleic acid and found in *F. polyphialidicum* (**Supplementary Figure 18**_C7_n9), or 279.2325@4.48, a potential isomer found in multiple strains including different *Fusarium* sp. strains, a *Colletotrichum* sp. strain (**Supplementary Figure 18**_C7_n10; 293.2475@5.28, annotated as the methyl ester, methyl alpha-linoleate, and shared between *Fusarium decemcellulare* and *L. venezuelensis* (**Supplementary Figure 18**_C7_n11). Similarly, octadecanoids were found in clusters specific to the leaf, *Curvularia* and *Fusarium* strains. Such fatty acids have been reported in *Lasiodiplodia* sp. and *Fusarium* sp. (Shahnazi et al., 2013; Salvatore et al., 2020).

In cluster C8, most of the unsaturated FA detected were annotated as methylated and oxygenated derivatives (**Figure S19**) and came from the fungal strains *C. eragrostridis* (A3, A14) and *L. venezulensis* (A2). Some of these features were shared with the plant leaves (**Supplementary Figures 19**_C8_n1–n3). According to literature, such derivatives have been found in *Curvularia* sp. and *Lasiodiplodia* sp. (Devi et al., 2006; Salvatore et al., 2020). (10*E*,12*Z*,15*Z*)-9-Hydroxy-10,12,15-octadecatrienoic acid methyl ester (n3) was found to be a defense substance in some plants against pathogenic fungi (Dong et al., 2000).

Unsaturated FA are ubiquitous in nature and essential components of eukaryotic cell membranes (Athenaki et al., 2018; He et al., 2020). Plyunsaturated fatty acids (PUFAs) are the precursors of oxylipins and their oxygenated metabolites. Oxylipins in plants serve as signaling molecules that mediate responses to biotic/abiotic stresses and developmental processes. In fungi, they are also linked to basic developmental processes (spores, germination, quorum sensing, and mycotoxins production) (Böttcher and Pollmann, 2009; Brodhun and Feussner, 2011).

Interestingly, fungal oxylipins were also detected among the shared features in the dataset.

**Figure 8:**
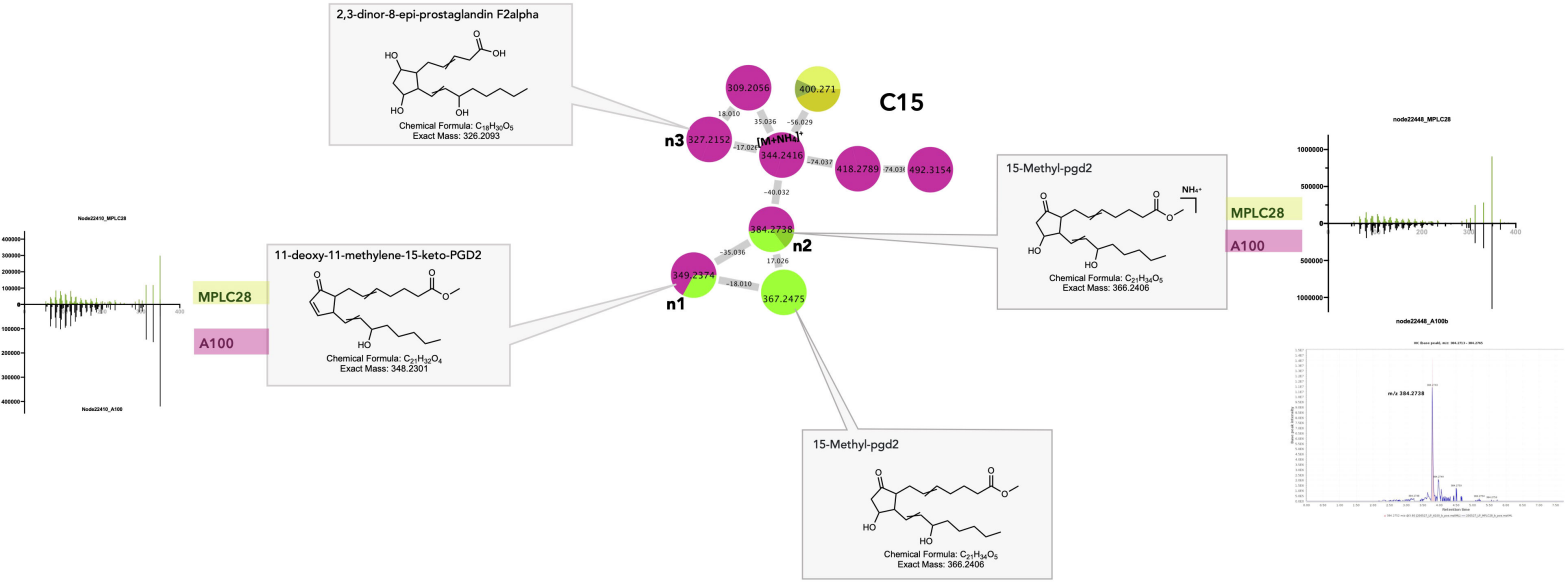
Selected annotations of cluster C15 from **Figure 6**, including various cyclic oxylipins. Annotated features n1and n2 are shared between the host leaf and the strain *Fusarium decemcellulare* A100 strain (for color code, see **Figure 6**). n3 corresponds to a specific cyclic oxylipin of strain A100, detected in culture, but not in the leaf. *m/z* 367.2475 corresponded to a specific cyclic oxylipin from the leaf. Representative MS/MS spectra of some annotated cyclic oxylipins and the single ion trace of the features across samples are displayed for shared features.

Cluster C15 (**Figure 6**_C15) contained several features annotated as eicosanoid (C20 PUFA) derivatives and prostaglandins (PGs). Two of the features annotated as PGs were detected to be shared between the *F. decemcellulare* strain A100 and the leaf (**Figures 8**_C15_n1, n2). Additionally, a cluster of cyclic oxylipins related to jasmonic acid (JA) and its derivatives could be detected mostly in the *Lasiodiplodia* A2 strain (e.g., **Supplementary Figure 2**_C8).

Eicosanoids are fungal oxylipins that are structurally similar to plant oxylipins and might be involved in signaling and regulating host immune responses, such as tolerance during colonization. Research has shown that PGs can be synthesized either *de novo* or using arachidonic acid in the host. Certain pathogenic fungi, such as *Bisifusarium dimerum*, employ prostaglandins as virulence factors to downregulate host immunity (Noverr et al., 2003). Several fungal species, such as *Fusarium oxysporum* and *Lasiodiplodia* sp., are known to secrete JA and its derivatives, which could play an important role in host–endophyte interactions (Miersch et al., 1999; Tsukada et al., 2010). Cyclic oxylipins have many physiological roles in plants, such as senescence, pathogen defense, and signaling processes (Schaller, 2001).

Oxylipins are regarded as interkingdom communication signals because of the striking similarity between the sets of oxylipins produced by plants and fungi, especially the lipoxygenase products, 9S-HPODE and 13S-HPODE.

Several studies have evidenced a “lipid language” between plants and fungi, involving PUFAs and oxylipins, which is likely to be conserved across multiple fungal species (Christensen and Kolomiets, 2011). Plant oxylipins can affect fungal secondary metabolism, whereas fungal oxylipins can mimic endogenous signaling molecules and influence processes in host tissues (Brodhun and Feussner, 2011). Certain plant pathogens can manipulate plant defense responses by mimicking the plant hormone JA (Wasternack and Kombrink, 2010). Conversely, certain fungi respond to plant oxylipins, which in turn influence their development (Calvo et al., 1999). Manipulation of host oxylipins by fungi is thought to be involved in enhancing symbiotic relationships; for example, by producing elicitors to induce JA biosynthesis and boosting the host’s resistance to fungal pathogens (Djonović et al., 2007).

The similarity of the metabolites identified here makes it difficult to conclude that the molecules found in the plant are remnants of the biosynthetic activity of fungi. However, because they are rather specific and detected mostly in fungal strains, their detection in the leaf could indicate the metabolic signature of these endophytes at the host level.

##### Shared dicarboxylic acids

**Figure 9:**
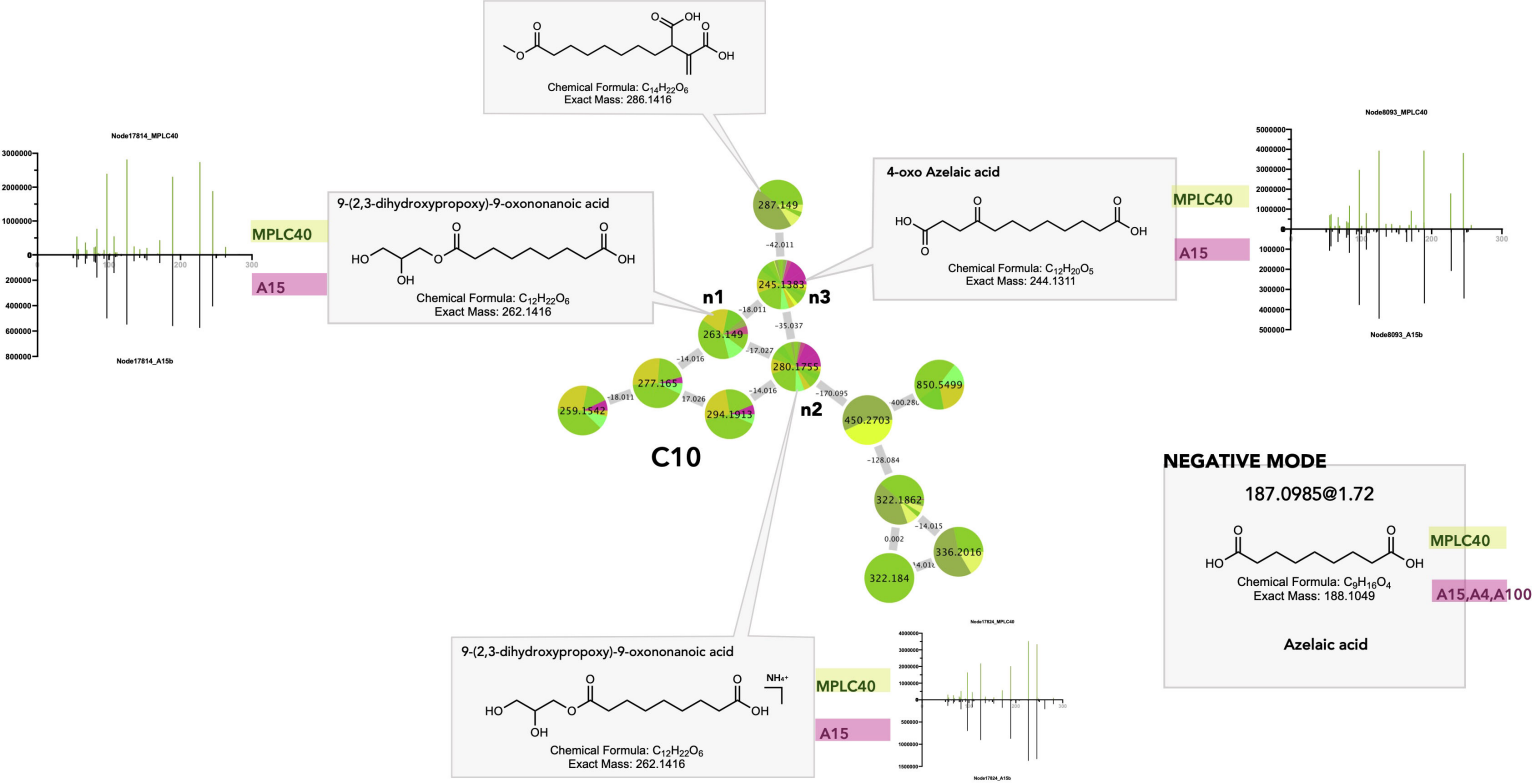
Selected annotations of cluster C10 from **Figure 6**, including various dicarboxylic acids. Annotated features n1, n2, and n3 were shared between the host leaf and the strain *Fusarium polyphialidicum* A15 (for color code, see **Figure 6**). *m/z* 287.149 corresponds to a specific dicarboxylic acid from the leaf. Azelaic acid was found to be a shared metabolite, as evidenced by the negative ion mode dataset. Representative MS/MS spectra of some annotated dicarboxylic acids and the single-ion trace of the features across samples are displayed for shared features.

Dicarboxylic acids and derivatives (FA and conjugate superclass) are particularly shared between plants and fungi. This is exemplified in clusters C10 and C11 (**Figures 6**_C10, 11). In C10, n1 and its ammonium adducts n2 and n3 were shared between multiple MPLC fractions and *Fusarium* sp. strains A15 and A100. Feature n3 was putatively annotated as 4-oxo-azelaic acid (4-oxo-AZA) (**Figure 9** C10_n3). In NI, the feature at 187.0985@1.72, shared between the *Fusarium* strains A4, A15, and A100 and multiple MPLC fractions, corresponded to the [M–H]^−^ of a molecule with MF C_9_H_16_O_4_ and was annotated as azelaic acid. This showed that azelaic acid and its 4-oxo derivatives were indeed shared between plants and fungi.

Dicarboxylic acids are produced by the oxidation of unsaturated fatty acids in higher plants and mammals (Mingrone and Castagneto, 2006). C_9_ dicarboxylic acid azelaic acid (AZA) is known as a defense messenger and a component of systemic acquired resistance (SAR), which is produced specifically after plant infection and travels through the plant via the vasculature to trigger immunity and defense signals (Jung et al., 2009). AZA and 4-oxo-AZA accumulate in mycorrhized plants as a local response to herbivory damage in leaflets and seem to be part of the leaf metabolome reprogramming after attacks, regulated by arbuscular mycorrhizal fungi. Moreover, 4-oxo-AZA has been isolated from *Fusarium* sp. (Sbrebryakov et al., 1970). Thus, its presence in the leaf might be the result of *Fusarium* strain production.

#### Shared features attributable to fungal strains

Through a detailed view of the metabolites produced by the fungi (see part *2*), the number of metabolites attributable to specific fungi could be identified and checked for their occurrence in the leaf samples.

##### Shared steroid derivatives

Steroid derivatives were identified as common components specific to plants (**Figures 4**_10–11) and fungi (**Figure 3**_1). Sterols appeared in several specific clusters (**Figures 6**_C12–C14) in the general MN, which seems logical since it is known that sterol biosynthesis is divergent in plant and fungi (Darnet et al., 2021). However, some features were shared, such as feature n1 in cluster C14 (**Supplementary Figure 20**_C14). The detection of sterols specific to fungi could be a trace of their membrane constituents, and thus, evidence of their occurrence in the leaf. Fungal sterols are also known to be involved in non-plant signal perception by plants, in both pathogenic and symbiotic interactions (Rodrigues, 2018).

##### Shared cytochalasan alkaloids

**Figure 10:**
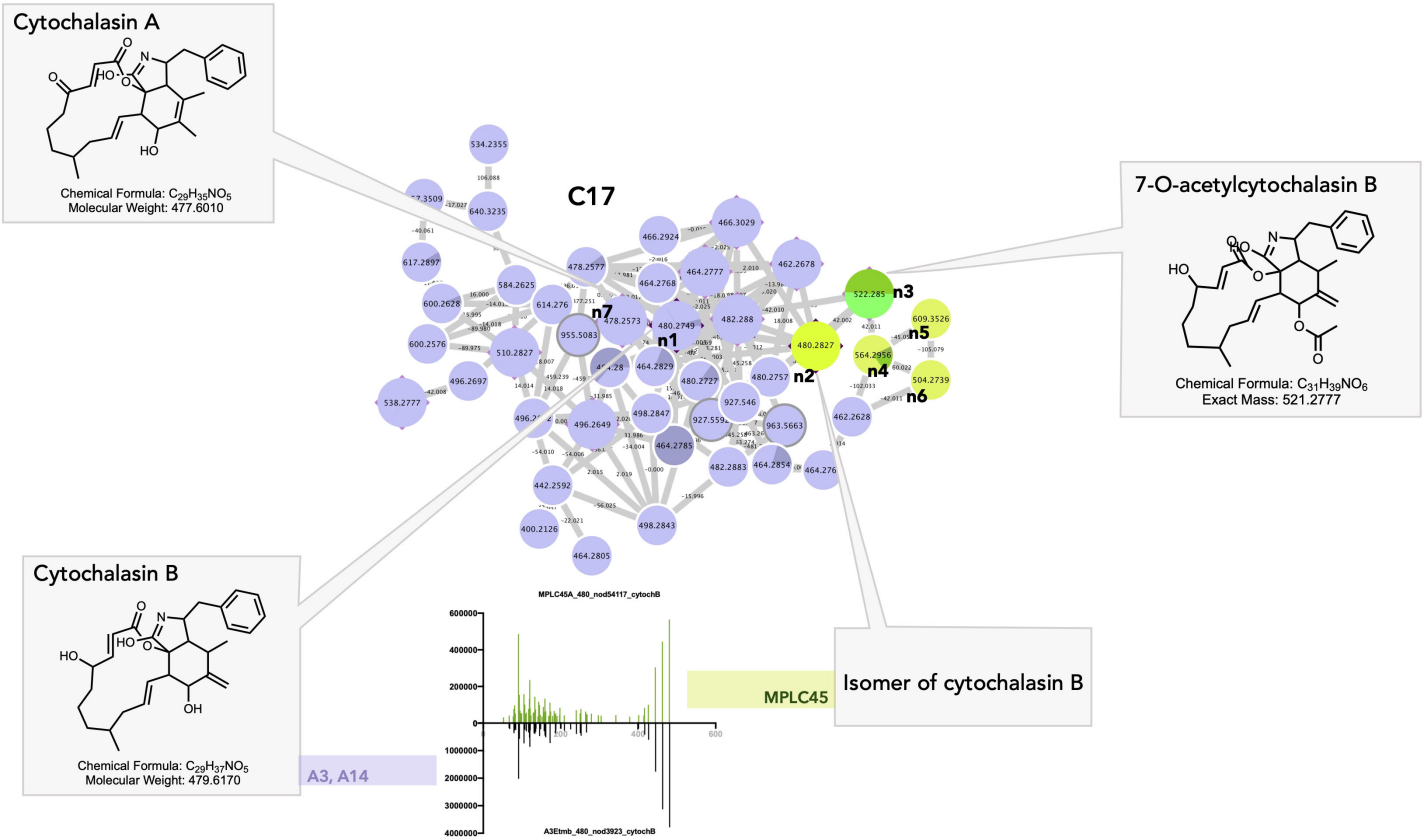
Selected annotations of cluster C17 from **Figure 6**, including various cytochalasin alkaloids. n7 corresponds to a specific cytochalasin alkaloid found only in the *Curvularia eragrostidis* strains A3 and A14. n3 to n6 correspond to the features of the leaf annotated as cytochalasin alkaloids. n1 and n2 corresponded to cytochalasin B isomers with very similar MS/MS spectra in both the leaf and *Curvularia eragrostidis* strains.

Cluster C17 gathers a group of cytochalasan derivatives in which the annotation of n1 was confirmed by injection of a cytochalasin B standard and propagated (**Figure 10**). Most of the features originating from the *Curvularia* strains A3 and A14 (**Figures 10**_C17_n2–n6) and cytochalasin B were isolated from *Curvularia lunata* (Wakker) Boedijn (Wells et al., 1981). Four associated nodes were obtained from the plant. Feature n2 at 480.2827@2.99 was detected in the plant only, but most probably corresponds most probably to an isomer of cytochalasin B. Feature n3 was also detected in the plant only and was annotated as 7-*O*-acetyl-cytochalasin B, which is only known to occur in fungi (*Phoma* sp. Sacc.) (**Figure 10**_C17_n3) (Capasso et al., 1991).

Cytochalasins are cell-permeable fungal metabolites (mycotoxins) isolated from *Xylaria* sp. Hill ex Schrank, *Phomopsis* sp. (Sacc.) Sacc., or from the Amazonian endophyte *Aspergillus* sp. P. Micheli ex Haller (Amaral et al., 2014; Chen et al., 2015; Feitosa et al., 2016; Chen et al., 2017). They have a wide range of biological activities and may be involved in the mediation of plant–fungi relationships, notably through the regulation of plant growth (cytochalasin H) (Cox et al., 1983) and the regulation of fungal growth and secretion (cytochalasin A, **Figure 10**_C17_n7) (Torralba et al., 1998). In particular, they act on cellular processes through their ability to bind actin filaments and inhibit their polymerization. Cytochalasins were found affect the outcome of attempted penetration of non-pathogenic fungi by acting on the polymerization of microtubules and actin filaments. Kobyashi et al., showed that a treatment of barley coleoptile with cytochalasin A allowed non-pathogens fungi including *Gloeosporium orbiculare* (Berk.) Berk.(taxon synonym *Colletotrichum lagenarium* (Pass.) Ellis & Halst.) and *Alternaria alternate* (Fr.) Keissl. to penetrate and form haustoria in coleoptile cells without affecting the stability and arrangement of microtubules. In untreated coleoptiles, non-pathogens always fail to penetrate plant cells (Kobayashi et al., 1997). Remarkably, a recent chemical investigation of a *Colletotrichum* sp. strain also isolated as an endophyte from *A. sciophilum* led to the isolation of cytochalasins, proving that they most likely represent the unique chemical marker of a fungal strain in this case (Barthélémy, 2019).

These findings demonstrate that certain fungal strains, as well as the leaf itself, exhibit detectable levels of cytochalasins. These compounds may have been used by certain endophytes to enter host plant cells, and their distinctive chemical signatures remain detectable even at the plant level. In addition to their antipathogenic properties, fungal cytochalasins may be present in the metabolome of plants to influence signaling pathways and facilitate interactions between the endophytes and their host plants.

##### Shared apocarotenoids

Cluster C18 was annotated as apocarotenoids ϵ-detected in different strains of *Fusarium*, *Curvularia*, and *Pestalotiopsis* as well as in the leaf (**Supplementary Figure 21**). Annotation of the feature n1 (**Supplementary Figure 21**_C18_n1) as 3-hydroxy-4,7-megastigmadien-9-one was confirmed because it was isolated from the plant. Apocarotenoids are carotenoid derivatives synthesized by the enzymatic action of carotenoid cleavage dioxygenase (CCD) in plants, algae, fungi, and bacteria (Nisar et al., 2015). They serve diverse key biological processes, including color and volatile attractants in plant–animal communications, and assume a significant signaling role in the interactions between plants and their environment, particularly in plant-microbial interactions (Walter et al., 2010). Some studies have shown that apocarotenoid biosynthesis is triggered in roots upon symbiotic arbuscular mycorrhizal colonization, resulting in the accumulation of cyclic C_13_ and linear C_14_ apocarotenoids such as 3-hydroxy-4,7-megastigmadien-9-one (n1), blumenols, and mycorradicins (Fiorilli et al., 2019). They have been proposed as foliar markers of AM symbiosis because of their transport to aboveground organs via the shoots, where they also accumulate. These molecules possess a moderate, rather than overly strong antimicrobial and growth-inhibiting properties, which may enable a milder defensive response to symbionts than to pathogens (Wang et al., 2018). Several studies have provided evidence that microbes, notably endogenous fungi isolated from boronia or marigold, are able to produce C_13_ norisporenoids, including 3-hydroxy-β-ionone (similar to n1), via the degradation of carotenoids such as lutein, facilitated by fungal enzymes (Sánchez-Contreras et al., 2000; Rodríguez-Bustamante et al., 2005; Khatri et al., 2010).

Interestingly, this study revealed that endophytic fungi can produce these compounds even when cultivated in isolation. Clusters of fungal carotenoids potentially serving as precursors were found in the same *Fusarium* strains (**Figures 6**_C19–20), indicating that these fungi likely possess the necessary substrates for biotransformation in the culture media.

## Discussion

The ability to detect common features in both fungal and host datasets within the global metabolomic dataset highlights the effectiveness of the workflow in generating data that allows for a comprehensive analysis of the co-occurrence of corresponding metabolites.

Overall, the findings indicated that only a small proportion of all detected features (3.7%) were common to both host leaf and fungal samples, despite the significant enrichment achieved through plant fractionation. This suggests that at the leaf level, the presence of fungi is barely detectable through their specific metabolomic signature obtained when the corresponding strains are cultivated. In general, the most common metabolites appear to align with those produced by fungi and plants. Therefore, it is difficult to determine whether this is indicative of a fungal presence in the leaf. Such discrimination would have only been possible with the analysis of a cultivated leaf devoid of its endophytic community. This was not the aim of this study because the focus was on monitoring the metabolome of the palm in its natural habitat.

This observation suggests that the endophytes present in the analyzed leaf samples may be metabolically inactive or show limited metabolic activity in this specific context, particularly concerning their specialized metabolites.

Nonetheless, as discussed, a subset of shared metabolites may be involved in the interplay between organisms (e.g., for signaling, infection establishment, and immune response). Several of these metabolites have been previously recognized as markers of fungal symbioses, particularly in plant roots, and our analysis reaffirms their significance in this context.

Research findings revealed that the expression of some metabolites and their associated genes increased upon endophyte presence and were linked to plant defense responses (oxylipins, unsaturated fatty acids, and JA), corroborating some of our results (Mejía et al., 2014).

As discussed above, oxylipins play a part in inter-kingdom cross-talk and might be involved in host-fungal communication because of the structural similarity between plant and fungal components (Christensen and Kolomiets, 2011). Fatty acids can be used by endophytes to influence the chemical and physical properties of leaf membranes, and in turn enhance the host’s robustness and resistance to pathogens while also reducing herbivore appearance. The production or modification of such metabolites can also be involved in the recognition of partner endophytes. For instance, cytochalasins can aid in colonization (Kobayashi et al., 1997), whereas GLs may contribute to differential recognition between symbionts and pathogens (Siebers et al., 2016).

Endophytes produce metabolites or induce the production of metabolites in the host, which are also directly involved in outcompeting undesired or pathogenic microbes by activating the plant’s immune and defense response through bioactive polyketides or sesquiterpenes, or dicarboxylic acids such as AZA and its derivatives (Mattoo and Nonzom, 2021).

On the other hand, prostaglandins can function as signaling molecules to downregulate host immunity and the presence of fungi in the host. The biosynthesis of metabolites such as C13 isoprenoids with moderate antipathogenic activity may allow for milder defensive measures against other symbiotic organisms. Ultimately, the metabolites produced by both the host and endophytes play a crucial role in facilitating fungal colonization and shaping endophyte communities. They allow endophytes to maintain asymptomatic growth while maintaining balanced antagonism with the host and other constituents of the microbiome (Schulz and Boyle, 2005). This delicate interplay of metabolites allows for nuanced and dynamic interactions between the host and various symbionts, ensuring a finely tuned coexistence and the health and functionality of the ecosystem.

Despite employing a consequent enrichment process, the detection of traces of specific metabolites *in folio* expressed *in vitro* proved to be challenging. This may be because these metabolites are not present in the leaves or exist in quantities below the detection limit of the analytical approach. They might also have a highly localized distribution within the leaf (Arnold et al., 2003) and be intertwined with a vast array of plant metabolites, thereby hampering their detection.

Another plausible explanation could be that metabolite production is low when fungi reside inside the leaves. Our previous studies have shown that potential endophyte communities in *A. sciophilum* appear to be uncompetitive. This was evidenced by the lack of anti-pathogen activity *in vitro* and through co-occurrence metagenomic studies (Donald et al., 2019). It could be assumed that this endophytic community of “old leaves” has established a balanced environment with its host, and might be metabolically “static” or not highly active, in contrast to a community constantly encountering host or exterior defense chemicals (Arnold et al., 2003).

Since some of the specific fungal metabolites and shared metabolites are detectable in the holobiont, it can be concluded that the endophyte community does contributes to its metabolome, at least in part. This does not prevent a contribution to the improved fitness of the plant. Indeed, a product present in small quantities can play a major biological role in plants. However, the chosen model (slow-growing palm in its natural environment) does not provide a clear explanation. As mentioned, studies involving plant material produced under perfectly sterile conditions are necessary to specifically assess the metabolome contribution of each endophytic fungus.

It must be recognized that the application of an untargeted metabolomic approach to the study of a complete holobiont remains extremely challenging, even after an extensive metabolite enrichment at the host level. An even more accurate detection of endophyte–host interplay at the molecular level may require more sensitive and/or targeted methods. Furthermore, it must be kept in mind that this represents only a part of the fungal endophytes, and other endophytes that are not cultivable *in vitro* have been missed.

Such studies could be complemented by using different models (other origins, different developmental stages), in which traceable systems can be used, target the producing organs, or work on differential inoculation in the host. Combinations of data with other omics tools (metagenomics, transcriptomics) to study associated biosynthetic genes, for instance, would help in understanding the underlying mechanisms involved in symbiosis (Lucaciu et al., 2019).

Our study provides a deep metabolomic survey of a long-lived holobiont in its natural habitat.

This contributes to extending the knowledge about the possible chemical interactions related to the symbiosis between the endophytic fungal community and the host using an advanced untargeted metabolomics workflow. The proposed approach can be adapted to the investigation of other host–microbe interactions. The results obtained shed light on how such challenging research topics can be addressed at the molecular level.

## Materials and methods

### Plant material

Healthy leaves of *A. sciophilum* were collected in August 2017 from the Nouragues National Nature Reserve at the Inselberg camp of the CNRS Ecological Research Station (French Guiana; 4°05’ N–52°41’W). Identification was carried out by Jerome Chave, a scientific director at the EDB (Evolution et Diversité Biologique) laboratory UMR5174, Toulouse. Leaves were sampled by randomly cutting 5 × 3 cm pieces of fresh leaflets and rachises from individual plants using sterile tweezers. Great care was taken to maintain sterile conditions throughout the process to avoid contamination with the environmental microbiome. The pieces were placed in Eppendorf tubes containing one-fourth Potato Dextrose Broth (PDB, Nutriselect™ Basic, Sigma-Aldrich, Germany) medium to allow the regrowth of fungi, as well as in tubes containing cetyl trimethylammonium bromide H6269 (CTAB, CAS: 57-09-0, Sigma-Aldrich, Germany) to preserve genetic material. All samples were then placed in a refrigerator to slow down the growth of fungi during the mission. Approximately 2 kg of fresh leaves from the same sampled individual *A. sciophilum* were collected and dried in an on-site oven for 48 h at 40°C (**Figure 1A**). The botanical material was monitored throughout the process and the treatment was stopped when the leaf was sufficiently dry. This procedure is necessary to allow sufficient drying in humid (tropical) environments.

### Isolation of endophytes

Isolation of the fungal foliar endophytic community of *A. sciophilum* and establishment of the collection were performed at the Agroscope in Changins (Federal Department of Economy, Education and Research, DEFR, Plant Protection Research Division). Each plant fragment stored in PDB tubes was carefully surface sterilized by washing under a stream of tap water for 3 h and then subjected to three successive 10-minute baths of sterile water under a laminar flow hood. Surface cleaning without an organic solvent was used to reduce the possible loss of diversity due to aggressive sterilization using alcoholic solutions. The surface-sterilized leaves were aseptically cut into small segments (0.5 cm^2^), placed individually in 9 cm Petri dishes containing potato dextrose agar medium (PDA, Potato Dextrose Agar, Sigma-Aldrich, Germany), amended with aureomycin (25 ppm.L^−1^) (to avoid the growth of bacteria and promote the growth of fungi only), cultured at room temperature, and inspected daily for the emergence of fungi. Each emerging hyphal tip was removed and transferred to fresh 9 cm Petri dishes containing PDA and aureomycin. Each hyphal fragment was isolated and grown separately. This yielded 15 strains of cultivable endophytes (**Figure 1B**). The isolated and selected strains were then individually cultured on a small scale in 9 cm Petri dishes containing PDA without aureomycin.

### Identification of fungal strains

Identification of the isolated strains was performed by extraction of ribosomal DNA (phenol/chloroform), PCR amplification of the ITS1F and ITS4 regions, and sequencing (Fasteris, Geneva, Switzerland). The obtained sequences were then submitted to BLAST^®^ in GenBank (GB, NCBI https://blast.ncbi.nlm.nih.gov/Blast.cgi) to identify the strain following Hofstetter et al. (2019). The specimens were integrated into the dynamic fungal library of Agroscope, whose content is available on the web (database Mycoscope: https://www.mycoscope.ch/) under reference numbers 1883–91, 1894–96, and 1981 in vials containing 5 ml of diluted PDB aqueous solution (1:4) at 4°C. The species identified for these strains are listed per strain in **Supplementary Table 1**.

### Culture and extraction of the isolated foliar fungal endophytes

Small-scale cultures were obtained by central inoculation of a 0.5 cm^3^ plug of agar (PDA medium) from the initial pure strain culture into the center of 9 cm Petri dishes containing PDA, repeated on 10 Petri dishes, and cultivated at room temperature until the plate was completely covered with the mycelium. The culture medium of the 10 cultures of each strain was then collected, cut into small pieces, mixed with ethyl acetate (EtOAc, Thommen-Furler, Bern, Switzerland), and extracted by maceration for 24 h under agitation at room temperature. The organic phase was recovered by vacuum filtration and washed three times with Millipore Corporation (Milli-Q) water (Elga Lab-Water, High Wycombe, UK). The organic (Et) and water (W) phases were dried under reduced pressure in a rotary evaporator (Büchi, Flawil, Switzerland) to yield crude mixtures. Agar from uninoculated PDA plates was treated in the same manner and used as a control.

### Plant extraction

Oven-dried leaves of *A. sciophilum* were ground into a thin powder. The ground material (760 g) was then successively extracted under maceration and agitation using a sequence of solvents of increasing polarity (hexane, ethyl acetate, methanol (Thommen-Furler AG, Bern, Switzerland, and MilliQ water) and concentrated under reduced pressure to yield four extracts: hexane (19.9 g), ethyl acetate (10.7 g), methanol (58.2 g), and water (15.3 g).

### Preparative MPLC-UV fractionation of the EtOAc leaf extract

The MPLC column (460 mm × 49 mm, 25 μm, i.d., Büchi) was packed with Silicagel 60 Å (40 μm –63 μm, Merck) as the stationary phase. The plant extract was introduced into the MPLC using a dry load cell: 8 g of extract was mixed with 27 g of stationary phase (Silicagel 60 Å, 40 μm –63 μm), at a ratio of 1:3 (extract:stationary phase) in 500 mL of HPLC grade EtOAc (Thermo Fisher Scientific, Waltham, USA), and then evaporated to dryness to give 35.13 g of a homogeneous powder in which 1 g of sand was added. The powder was then placed into a dry load cell (special aluminum column of 11.5 cm × 2.7 cm, connecting the pumps and the MPLC column, allowing a higher pressure than with the usual glass precolumn (Challal et al., 2015)) between of two layers of sand. Optimized normal-phase analytical HPLC (NP-HPLC) conditions were geometrically transferred to a preparative MPLC system (Guillarme et al., 2008). Analytical NP-HPLC analyses were conducted as follows: Scorpio Si 60 Å column (250 mm × 4.6 mm, 15 μm spherical, BGB); solvent system hexane (A), EtOAc (B); separation with a gradient step from 10% to 25% of B over 2 min, isocratic step at 25% of B over 23 min, gradient step from 25% to 60% of B over 20 min, and 60% to 100% of B over 15 min, held for 10 min; flow rate fixed at 1 mL/min; UV detection recorded at 210 nm, 254 nm, 280 nm, and 366 nm. The preparative MPLC conditions were as follows: MPLC column (460 mm × 49 mm, 25 μm, i.d.) packed with Silicagel 60 Å (40 μm–63 μm); solvent system hexane (A), EtOAc (B); separation with a gradient step from 10% to 25% of B over 14 min, isocratic step at 25% of B over 109 min, gradient step from 25% to 60% of B over 94 min, and 60% to 100% of B over 71 min, held for 48 min; and flow rate fixed at 40 mL/min. UV detection was performed at 254 nm, 280 nm, 366 nm, and 560 nm. The separation yielded 50 fractions of 250 mL, which were dried under reduced pressure using a rotary evaporator. The MPLC fractions were then monitored by UHPLC-UV-ELSD-QDa analyses of the MPLC fractions using an Acquity UHPLC system interfaced to a QDa mass spectrometer (Waters^®^, Milford, USA) and an electrospray ionization (HESI-II) source. Chromatographic separations were performed as follows: Acquity BEH C18 column (2.1 mm × 50 mm; 1.7 μm); solvent system water (A) and acetonitrile (B), both with 0.1% FA; separation with a linear gradient from 5% to 100% of B in 7 min and a 1 min isocratic step at 100% of B; flow rate fixed at 600 μL/min; temperatures autosampler and column oven at 10°C and 40°C. UV data were acquired from 200 nm to 600 nm; ELSD temperature at 45°C; pressure at 3.5 bar; gain at 8. Mass data were obtained by fully automated acquisition at 10 Hz for *m/z* values between 100 and 1,000. Data acquisition, instrument control, and data processing were performed using Masslynx^®^ software (Waters^®^).

### Mass spectrometry analysis

#### UHPLC-HRMS/MS metabolite profiling

Chromatographic separation was achieved on a Waters Acquity UPLC system interfaced with a Q-Exactive Focus mass spectrometer (Thermo Scientific, Bremen, Germany) using a heated electrospray ionization (HESI-II) source. Thermo Scientific Xcalibur 3.1 software was used for instrument control. LC separation was conducted as follows: Acquity BEH C18 column (2.1 mm × 50 mm; 1.7 m, Waters); mobile phase, water (A), acetonitrile (B) with 0.1% formic acid; flow rate fixed at 600 μL/min; injection volume 4 μL; linear gradient from 5% to 100% of B in 7 min and isocratic at 100% of B for 1 min. The optimized HESI-II parameters were as follows: source voltage, 3.5 kV (pos), 4 kV (neg); sheath gas flow rate (N_2_), 55 units; auxiliary gas flow rate, 15 units; spare gas flow rate, 3.0; capillary temperature, 275°C (pos), 320°C (neg); S-Lens RF Level, 45. The mass analyzer was calibrated by direct injection using a mixture of caffeine, methionine-arginine-alanine-acetate (MRFA), sodium dodecyl sulfate, sodium taurocholate and Ultramark 1621 in an acetonitrile/methanol/water solution containing 1% formic acid.

Data-dependent MS/MS events were performed on the three most intense ions detected in the full-scan MS (Top3 experiment). The MS/MS isolation window width was 1 Da and the normalized collision energy was set to 15, 30, and 45 units. In the data-dependent MS/MS experiments, full scans were acquired at a resolution of 35,000 FWHM (at *m/z* 200) and MS/MS scans at 17,500 FWHM, both with an automatically determined maximum injection time. After acquisition in an MS/MS scan, precursor ions were placed in a dynamic exclusion list for 2.0 s. Detection was performed in the positive ion mode (PI) and negative ion mode (NI) with an *m/z* scan range of 130–1,950.

Leaf MPLC fractions and EtOAc crude extract (48 samples) were analyzed in the first part of the series, fungal extracts (34 samples) in the second part of the series, with a thorough cleaning step with blank samples to ensure no interference between the 2 series. Samples were analyzed in triplicate and in random order (48 × 3 + 30 × 3 = 234 samples). Quality controls (QC, equal mixture of all the samples for each series) and blank samples (injection solvent only) were included every 10 runs. Blank PDA samples (extracted from uninoculated culture media) were also added. In addition, five analyses of both the blank and QC samples were performed before and after the analyses of both sets. Analytical standards and molecules isolated from some of the strains individually investigated in our laboratory were analyzed separately.

#### UHPLC-HRMS/MS data processing

The UHPLC-HRMS/MS data were converted from.RAW (Thermo) standard data format into.mzXML format using MS conversion software (ProteoWizard package (Chambers et al., 2012)). The converted files were processed using MZmine (v2.51) (Pluskal et al., 2010), and all previously described samples were processed in the same batch of MZmine analyses to allow for comparison of MS data. Mass detection was performed using a centroid mass detector with a noise level set at 4E5 (1E4 NI) for MS level 1 and 0 for MS level 2. The ADAP chromatogram builder was employed and set to a minimum group size of five scans, a minimum group intensity threshold of 4E5 (1E4 NI), the minimum highest intensity of 4E5 (1E4 NI) and an *m/z* tolerance of 8.0 ppm. The wavelets ADAP algorithm was used for chromatogram deconvolution with the following settings: intensity window single to noise (S/N) as S/N estimator with S/N threshold set at 15 (20 NI), a minimum feature height at 4E5 (1E4 NI), coefficient area threshold at 150 (100 NI), peak duration range 0.02 to 0.9 min (0.02–0.8 NI), wavelet range of 0.01 min to 0.04 min. Isotopes were detected using the isotope peak grouper with *m/z* tolerance set at 8.0 ppm, RT tolerance at 0.02 min (absolute), maximum charge of 2, and representative isotope as the most intense. Each feature list was filtered before alignment to remove duplicates using the duplicate peak filter with RT tolerance at 0.03 min and *m/z* tolerance at 8 ppm, and to keep only features with an associated MS2 scan using the peak list row filter. Peak alignment was performed using the join aligner method with an *m/z* tolerance of 10 ppm, absolute RT tolerance of 0.04 min, weight for *m/z* and RT at 10, and a weighted dot-product cosine similarity of 0.7. Finally, a local spectral database (DB) search identification was conducted using a custom DB (.msp file) containing MS data of standards and previously isolated molecules ran in the same batch of analyses, with *m/z* tolerance at 20 ppm and weighted dot-product cosine similarity at 0.8. The aligned feature list is exported using the GNPS export module.

#### Feature table processing

A unique feature table of the 246 samples and their blanks was obtained. The table was filtered by subtracting peaks from the blank and the media and by keeping only the features detected in the three replicates of in at least one sample. To establish the co-occurrence of a given retained feature in other samples, its detection had to be recorded at least in two replicates. The intensities were then normalized by the feature (sum of the intensities in the different samples for each ion = 1). The average intensity of three replicates was calculated for each feature. This filtering process led to the discarding of approximately 33,205 features out of 57,301 from the initial table for positive ionization (PI) analyses and 8,775 out of 19,567 for negative ionization (NI) analyses. This resulted to a final feature list: general dataset = 24,096 features (PI) and 10,792 (NI). This feature list was then filtered based on the sample origin from all fungal samples on one side and all leaf samples on the other, to yield two feature lists: fungal dataset = 11,072 (PI), 5,982 (NI) and host dataset = 13,884 (PI), 5,503 (NI). The.mgf files associated with each dataset were extracted to allow molecular network processing. Mass spectrometry data were deposited in the MassIVE public repository (n° MSV000088516).

#### Molecular network generation

To maintain the RT and exact mass information and to allow for isomer separation, feature-based MN were created using the.mgf file resulting from the MZmine pretreatment steps detailed above (FBMN). The spectral data were uploaded to the GNPS MN platform. An MN was created where edges were filtered to have a cosine score above 0.7 and more than six matched peaks. Furthermore, edges between two nodes were kept in the network if, and only if, each of the nodes appeared in each of the top 10 most similar nodes. Finally, the maximum size of a molecular family was set to 100, and the lowest scoring edges were removed from the molecular families until the molecular family size was below this threshold. The spectra in the network were then searched against the GNPS spectral libraries (Wang et al., 2016). All matches kept between the network spectra and library spectra were required to have a score above 0.7 and at least six matched peaks. The output was visualized using Cytoscape software v3.8.0 (Shannon et al., 2003). The different GNPS job parameters and the resulting data are available at the following addresses:

PI fungal dataset: https://gnps.ucsd.edu/ProteoSAFe/status.jsp?task=28f944e757d54755948824ca55e4a601
PI host dataset: https://gnps.ucsd.edu/ProteoSAFe/status.jsp?task=75c277bd53a341ff826f63ea4d1a0e3e
PI general dataset: https://gnps.ucsd.edu/ProteoSAFe/status.jsp?task=250536f4cb3e4f159e5ef67a3d024fac
NI fungal dataset: https://gnps.ucsd.edu/ProteoSAFe/status.jsp?task=b28bfcec6019409ca75c6aae0348eccf
NI host dataset: https://gnps.ucsd.edu/ProteoSAFe/status.jsp?task=d87595a93200444286531b89e1ffb692
NI general dataset: https://gnps.ucsd.edu/ProteoSAFe/status.jsp?task=b786792e271d46bbb6816853f69eeb25

#### Class annotation for the whole dataset

Filtered features detected in the PI in the general dataset were annotated using a computational approach integrating SIRIUS (molecular formula), CSI:fingerID (probabilistic molecular fingerprint by machine learning substructure prediction and *in silico* annotation), and CANOPUS (systematic class annotation) (Dührkop et al., 2015; Dührkop et al., 2019; Dührkop et al., 2021). SIRIUS analyzes both the isotope and fragmentation patterns to determine the molecular formula of the measured precursor ions. Furthermore, it uses CSI:fingerID to predict a molecular fingerprint from the related MS/MS spectrum and fragmentation tree and to propose putative annotations by searching molecular structure databases. Finally, CANOPUS predicts compound chemical classes using the NPClassifier taxonomy from the molecular fingerprint generated by CSI : FingerID (Dührkop et al., 2015; Dührkop et al., 2019; Dührkop et al., 2021).

#### Taxonomically informed metabolite annotation

The spectral files (.mgf) and attribute metadata (.clustersummary) obtained after the MN step were annotated first at the MS1 level by matching the precursor *m/z* with all the potential *m/z* values of the most common adducts of the compounds reported in the taxonomic family of the analyzed sample, followed by the MS/MS level against a custom version of the LOTUS-ISDB (in-house database containing the *in silico* fragmentation spectra of all the compounds present in the DNP and LOTUS databases) complemented with structure–organism pairs from the Dictionary of Natural Products DNP (Zenodo) (Allard et al., 2016; Rutz et al., 2019). The following parameters were used: spectral match parameters: parent mass tolerance 0.01 Da, MS/MS tolerance 0.01 Da, minimum cosine score 0.2, minimum peaks, 6. Spectral match of MS/MS spectra against the database provided a list of 50 chemical structure candidates for every feature. The candidates were re-ranked by taxonomic reweighting after ponderation of their spectral score, which was inversely proportional to the taxonomic distance between the biological source of the candidate and that of the analyzed sample(s) (e.g., the fungal strain) in which the feature was detected (Rutz et al., 2019). Based on the MN topology, a consensus chemical class at each NPclassifier level (Kim et al., 2021) (pathway, superclass, class) was returned for every node, and annotations were re-ranked accordingly. Finally, the top three candidates were retained.

Interactive plot GitHub repository: leonie74/leonie74.github.io.

## Data availability statement

The original contributions presented in the study are included in the article/**Supplementary Files**. Further inquiries can be directed to the corresponding author/s.

## Author contributions

LP: Conceptualization, Data curation, Formal analysis, Investigation, Methodology, Validation, Visualization, Writing – original draft, Writing – review & editing. AG: Data curation, Formal analysis, Software, Writing – review & editing. SV: Investigation, Writing – review & editing. NL: Investigation, Methodology, Resources, Writing – review & editing. AR: Software, Writing – review & editing. P-MA: Software, Writing – review & editing. LM: Formal analysis, Writing – review & editing. EF: Supervision, Writing – review & editing. JC: Resources, Supervision, Writing – review & editing. VE: Conceptualization, Methodology, Writing – original draft, Writing – review & editing. DS: Methodology, Supervision, Writing – review & editing, Funding acquisition. KG: Conceptualization, Methodology, Resources, Supervision, Writing – review & editing. J-LW: Conceptualization, Funding acquisition, Methodology, Project administration, Resources, Supervision, Validation, Writing – original draft, Writing – review & editing.

## Footnotes

1
https://leonie74.github.io.
