# Supplementary material for: Comparative metabolomic study of fungal foliar endophytes and their long-lived host Astrocaryum sciophilum: a model for exploring the chemodiversity of host-microbe interactions
Source: Front Plant Sci. 2023 Dec 19;14:1278745. doi: 10.3389/fpls.2023.1278745 (PMC10768666; doi:10.3389/fpls.2023.1278745)
Supplement: Supplementary file 3 [file DataSheet_3.pdf]

Supplementary Material

Comparative metabolomics of fungal foliar endophytes and their long-lived host *Astrocaryum sciophilum*: a model for exploring the chemodiversity of host-microbe interactions.

Leonie Pellissier[1,2]*, Arnaud Gaudry[1,2], Salomé Villette[1,2], Nicole Lecoultre[3], Adriano Rutz[1,2], Pierre-Marie Allard[1,2,4], Laurence Marcourt[1,2], Emerson Ferreira Queiroz[1,2], Jérôme Chave[5], Didier Stien[6], Katia Gindro[3], Jean-Luc Wolfender[1,2]*

*** Correspondence:**

Léonie PELLISSIER lpellissier@invaio.com

Jean-Luc WOLFENDER Jean-Luc.Wolfender@unige.ch

A1	*Neopestalotiopsis ellipsospora* (Maharachch. & K.D. Hyde) Maharachch., K.D. Hyde & Crous
A2	*Lasiodiplodia venezuelensis* T. Burgess, Barber & Mohali
A3	*Curvularia eragrostidis* (Henn.) J.A. Mey.
A4	*Fusarium concolor* Reinking
A5	*Neopestalotiopsis clavispora* (G.F. Atk.) Maharachch., K.D. Hyde & Crous
A6	*Fusarium concolor* Reinking
A7	*Neopestalotiopsis clavispora* (G.F. Atk.) Maharachch., K.D. Hyde & Crous
A8	*Diaporthe terebinthifolii* R.R. Gomes, Glienke & Crous
A9	*Mucor* P. Micheli
A13	*Trichoderma lentiforme* (Rehm) P. Chaverri, Samuels & F.B. Rocha
A14	*Curvularia eragrostidis* (Henn.) J.A. Mey.
A15	*Fusarium concolor* Reinking
A16	*Colletotrichum* Corda
A17	*Colletotrichum paranaense* C.A.D. Bragança & Damm
A100	*Albonectria rigidiuscula* (Berk. & Broome) Rossman & Samuels

Table S1. Foliar endophytic fungi collection strains with their identifications

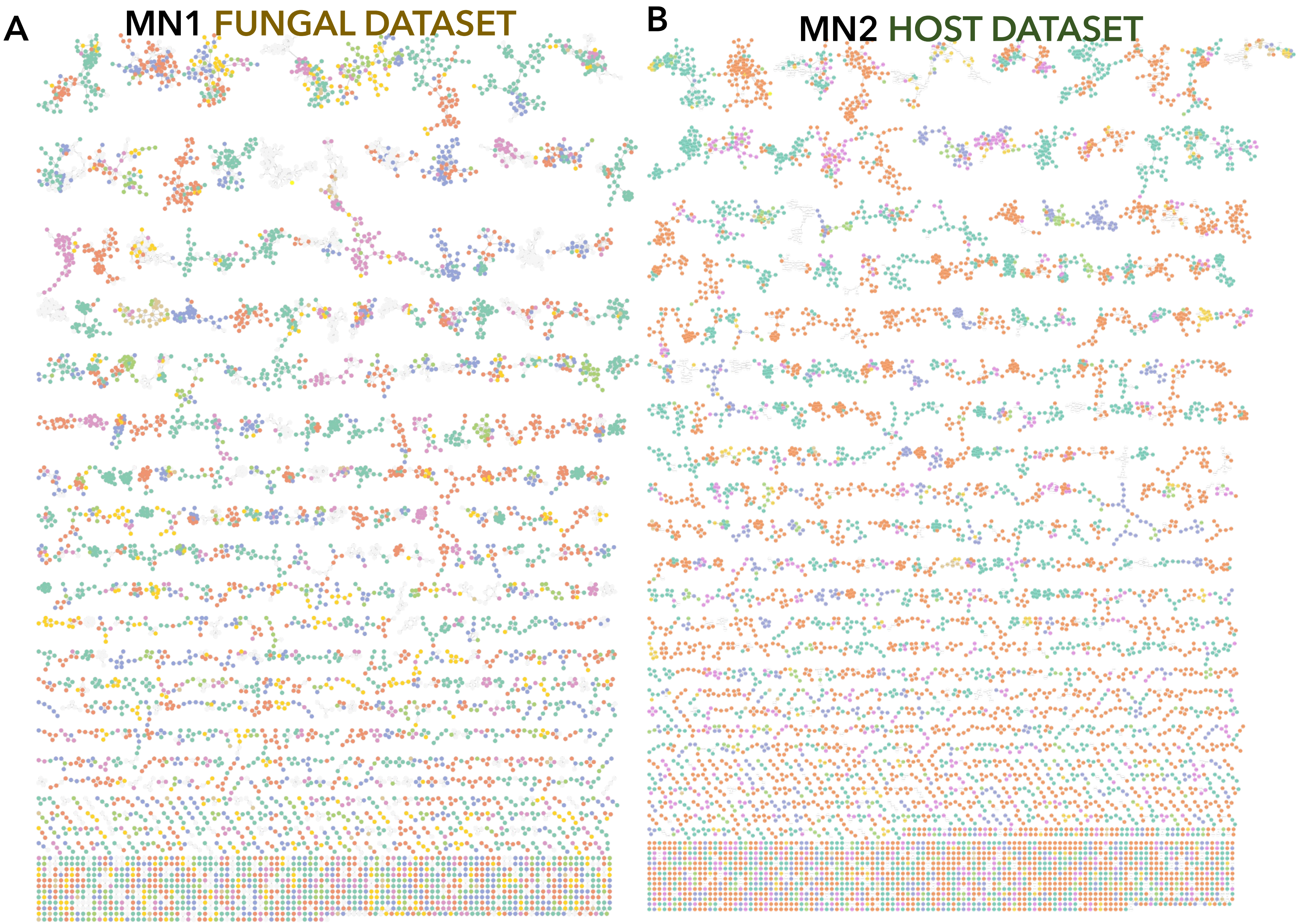

Figure S1. Molecular network on the fungal (A, MN1) and the host (B, MN2) datasets, each chemical pathway is assigned to a colour to map the repartition of chemical pathway and highlight the structural type for each node.

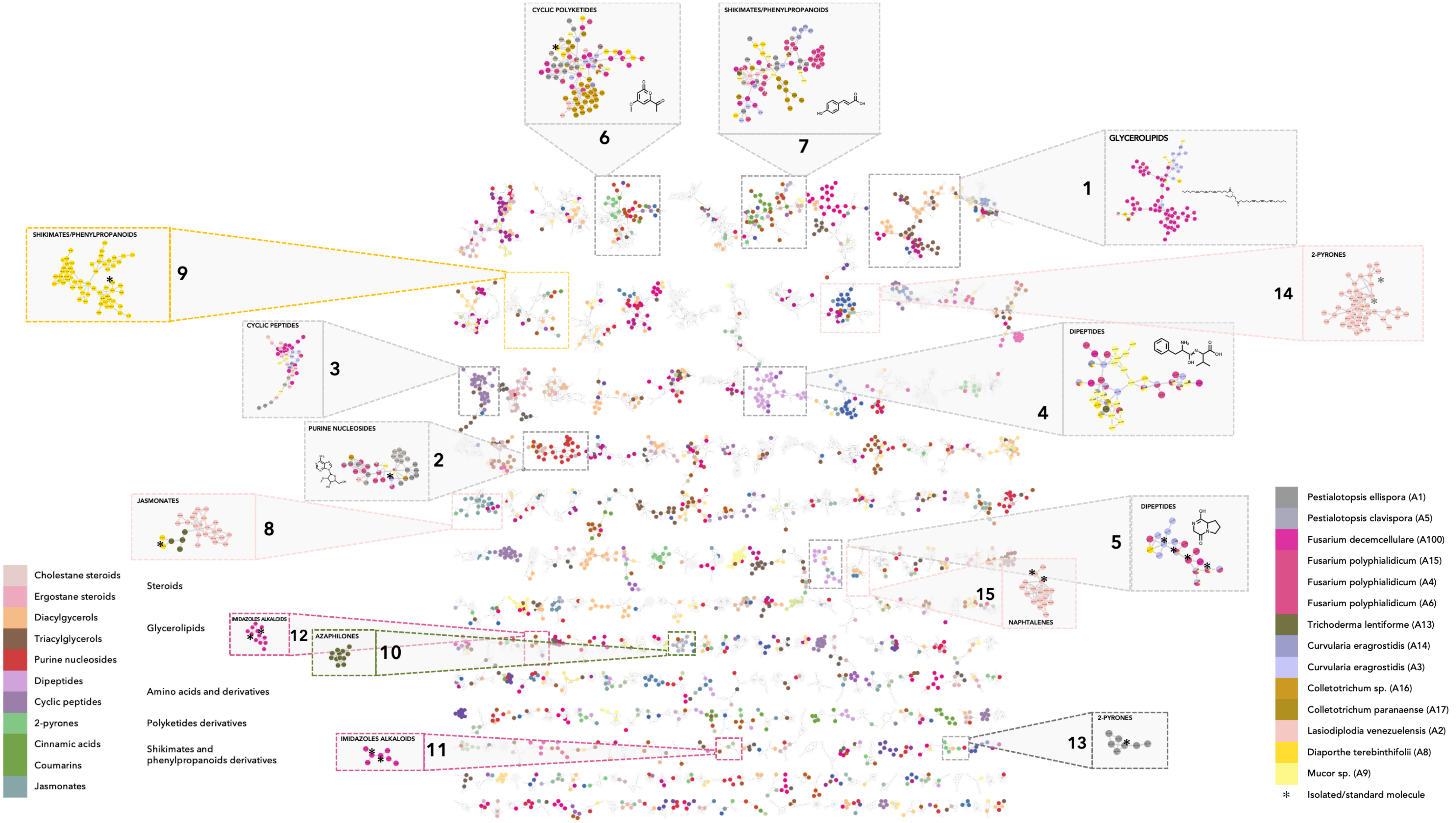

Figure S2. MN on the fungal dataset with chemical class colour mapping, with cluster of interested highlighted in zoomed squares, with taxonomical colour mapping.

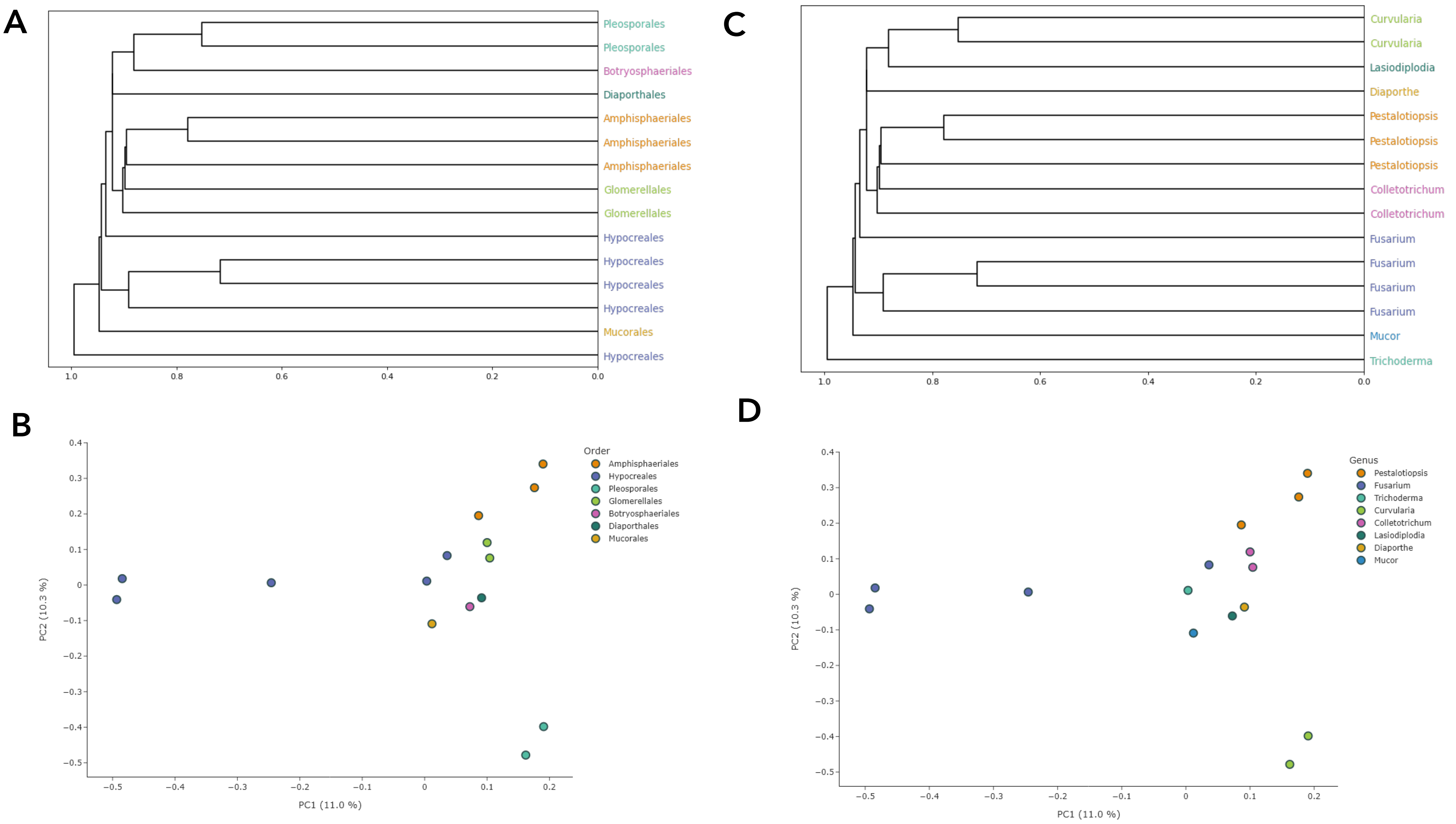

Figure S3. PCoA (Braycurtis dissimilarity metrics) at the order level (A) and the genus level (B). HCA (single linkage method) at the order level (C) and the genus level (D).

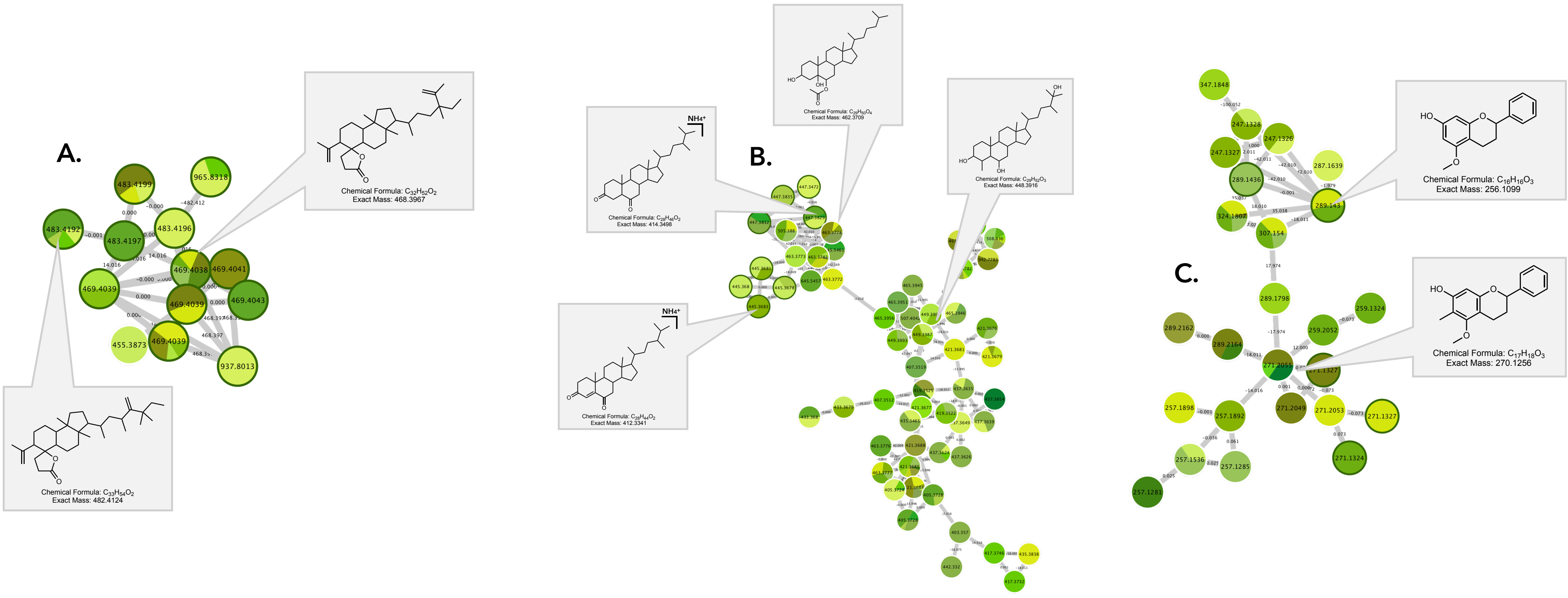

Figure S4. Clusters representing (A) lanostane triterpenoids; (B) cholestane steroids; (C) flavonoids

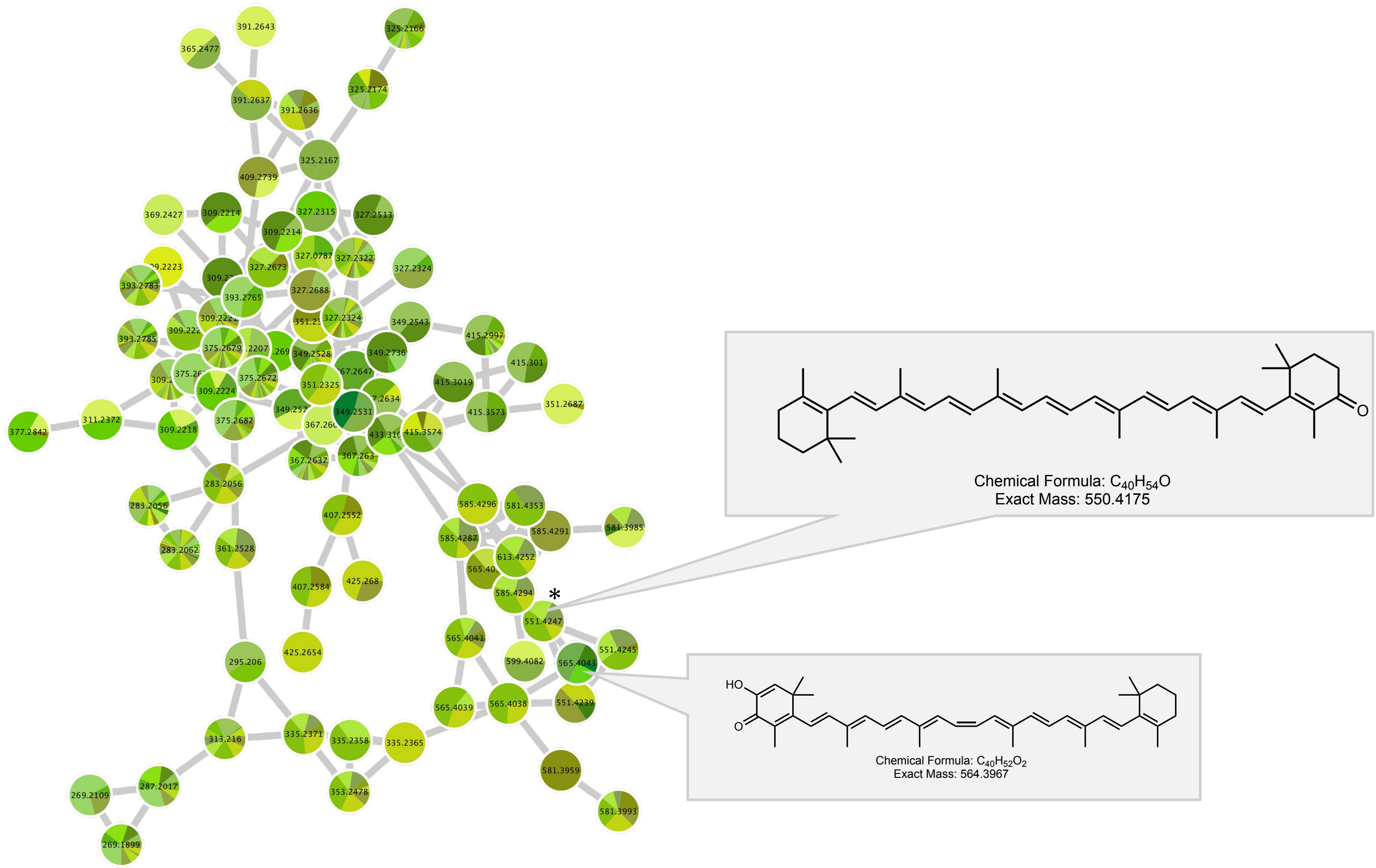

Figure S5. Cluster representing carotenoids.

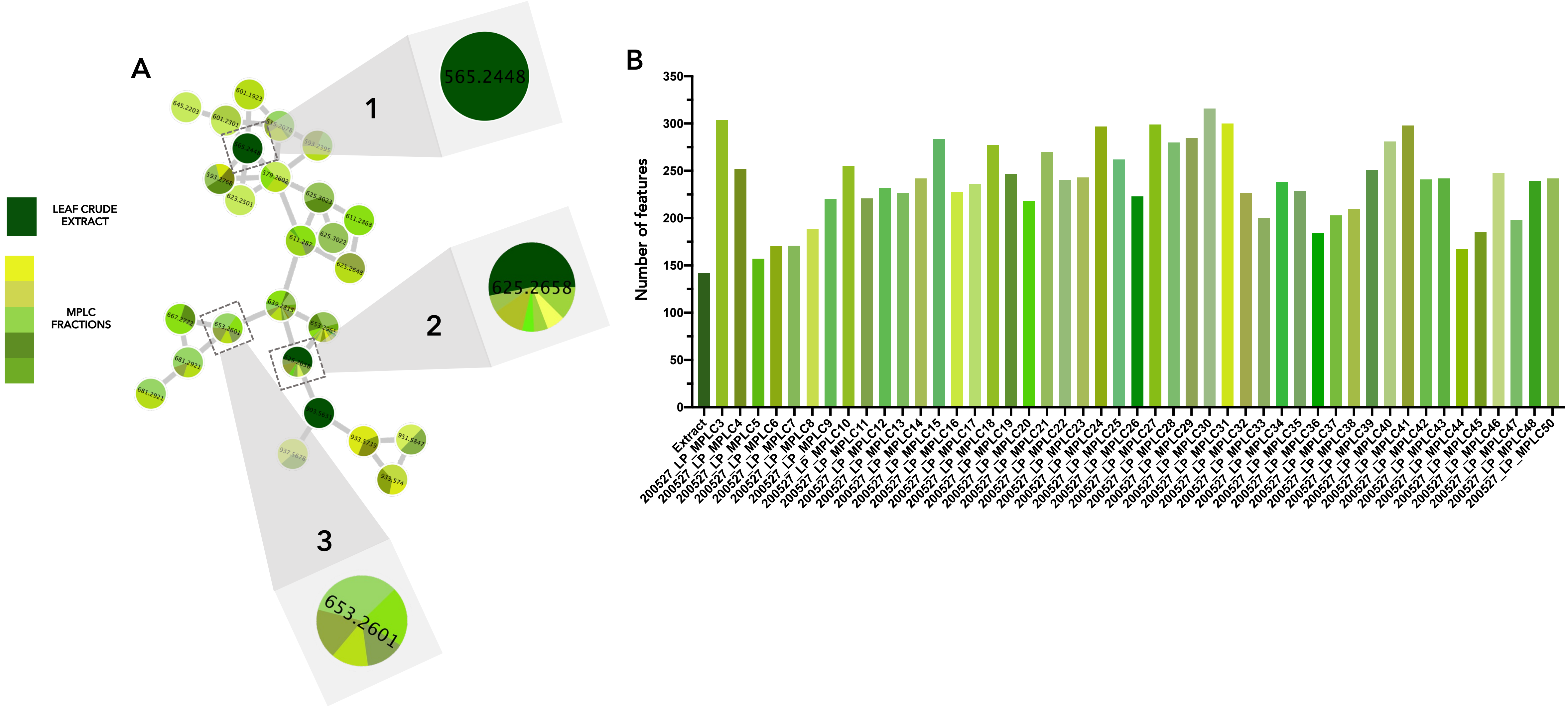

Figure S6. (A) Example of a cluster with repartition of the nodes according to their detection (1) in the raw extract, (2) only in the MPLC fractions or (3) in both, (B) Number of features detected in the different samples: crude extract and MPLC fractions.

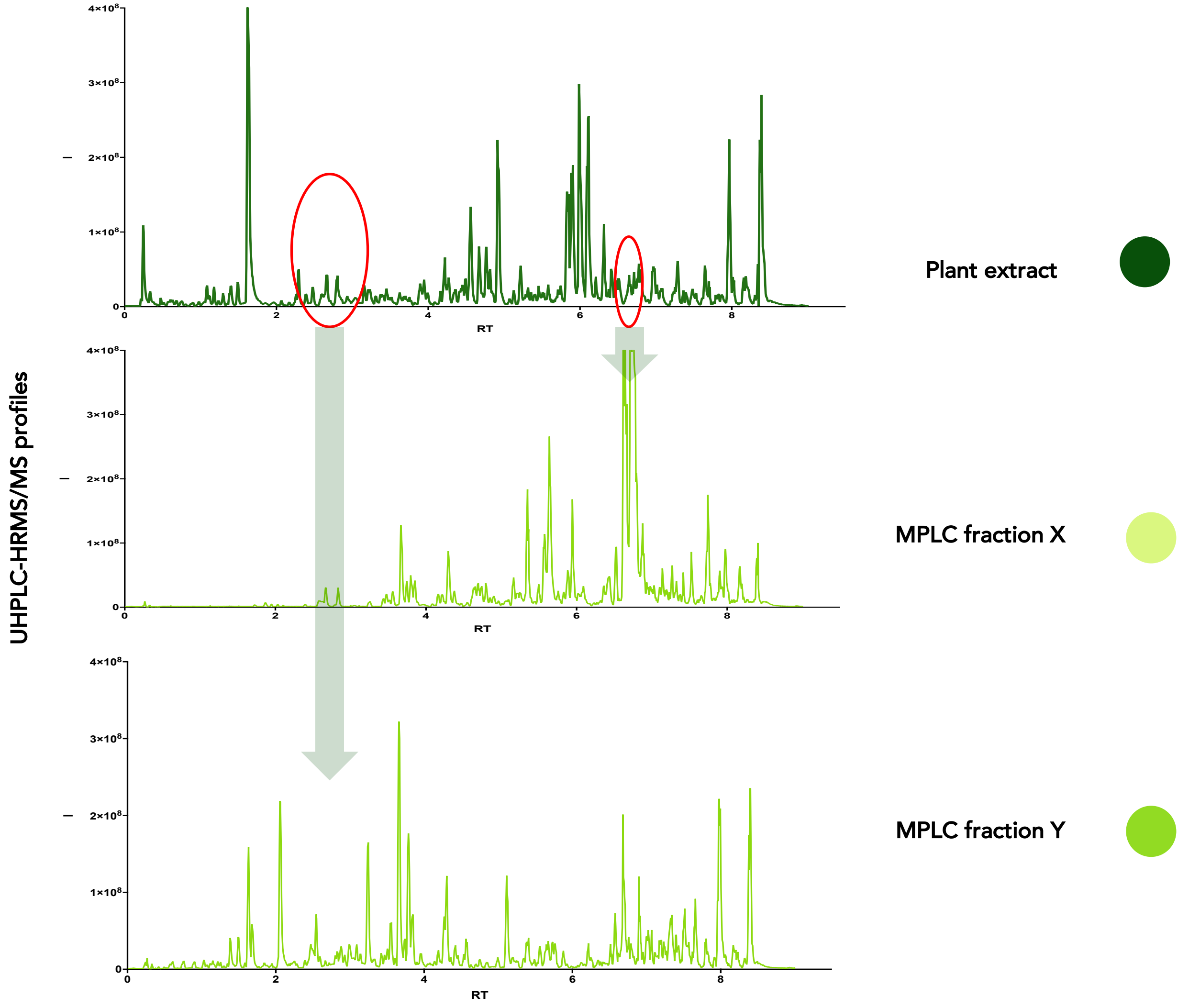

Fig S7. Comparison of UHPLC-HRMS profiles between the plant crude EtOAc extract and example of MPLC fractions, showing the efficacy of enrichment through preparative fractionation.

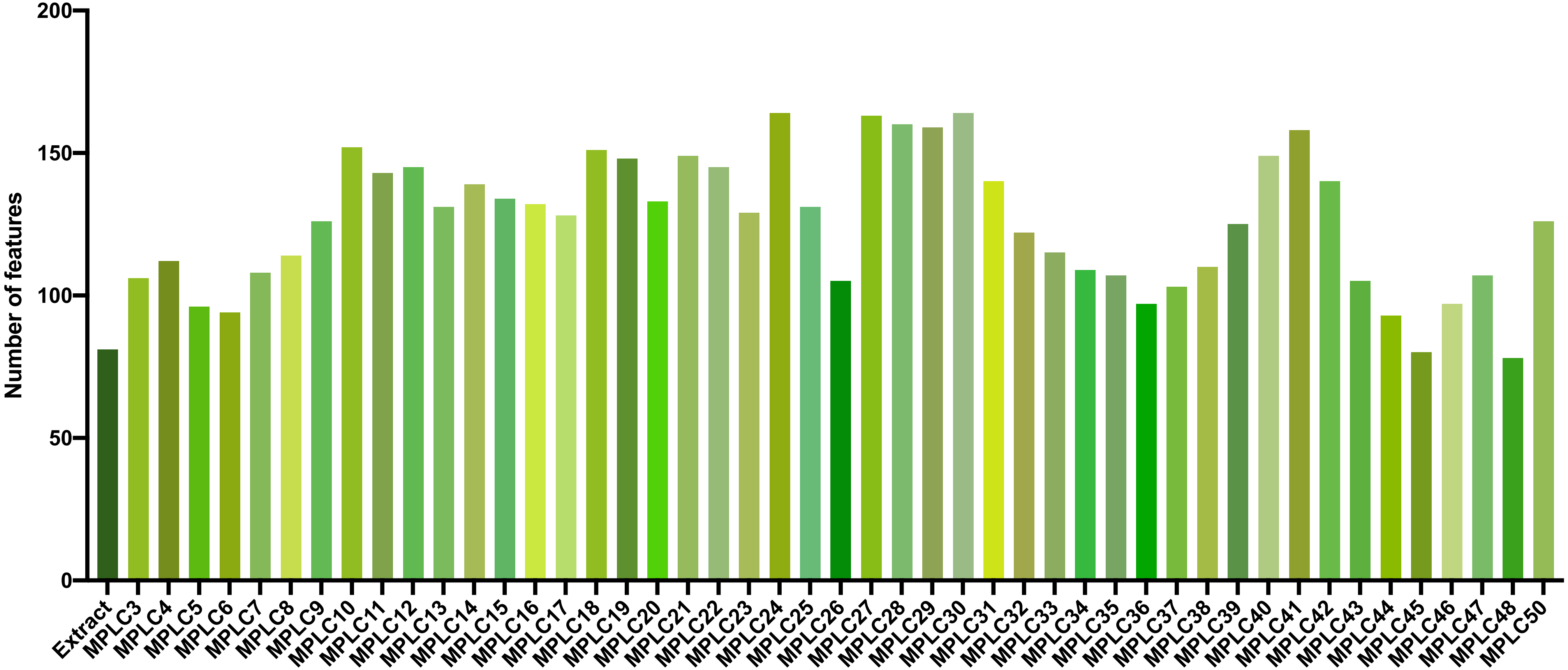

Figure S8. Number of features per leaf sample: crude EtOAc extract and MPLC fractions (PI).

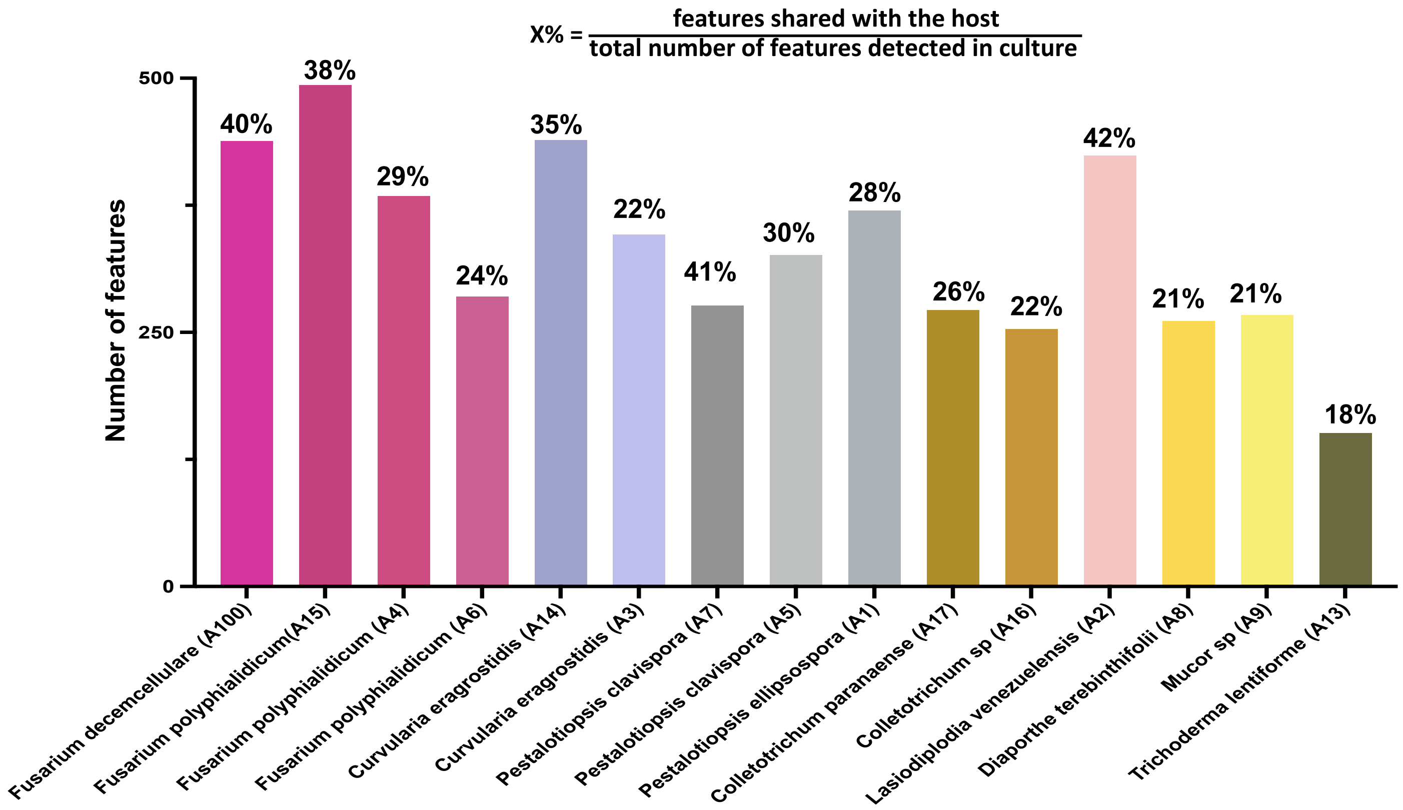

Figure S9. Number of features shared with the host per fungal strain and percentage of the metabolome that is shared with the host, for each strain (PI+NI).

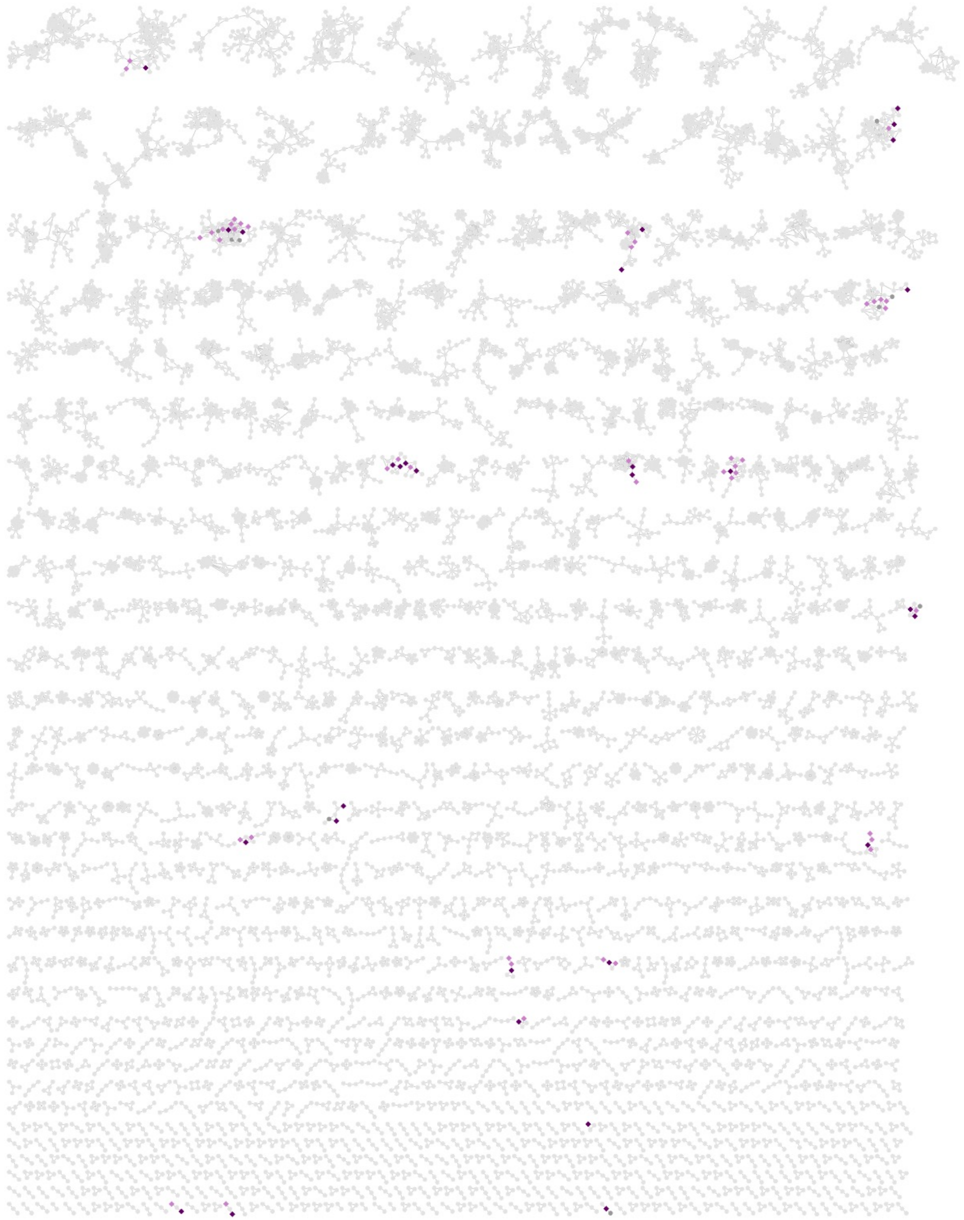

Figure S10. Molecular network on the general dataset, features identified using standards and previously isolated molecules highlighted in dark violet, and example of propagation of the annotation to neighbouring features in lilac.

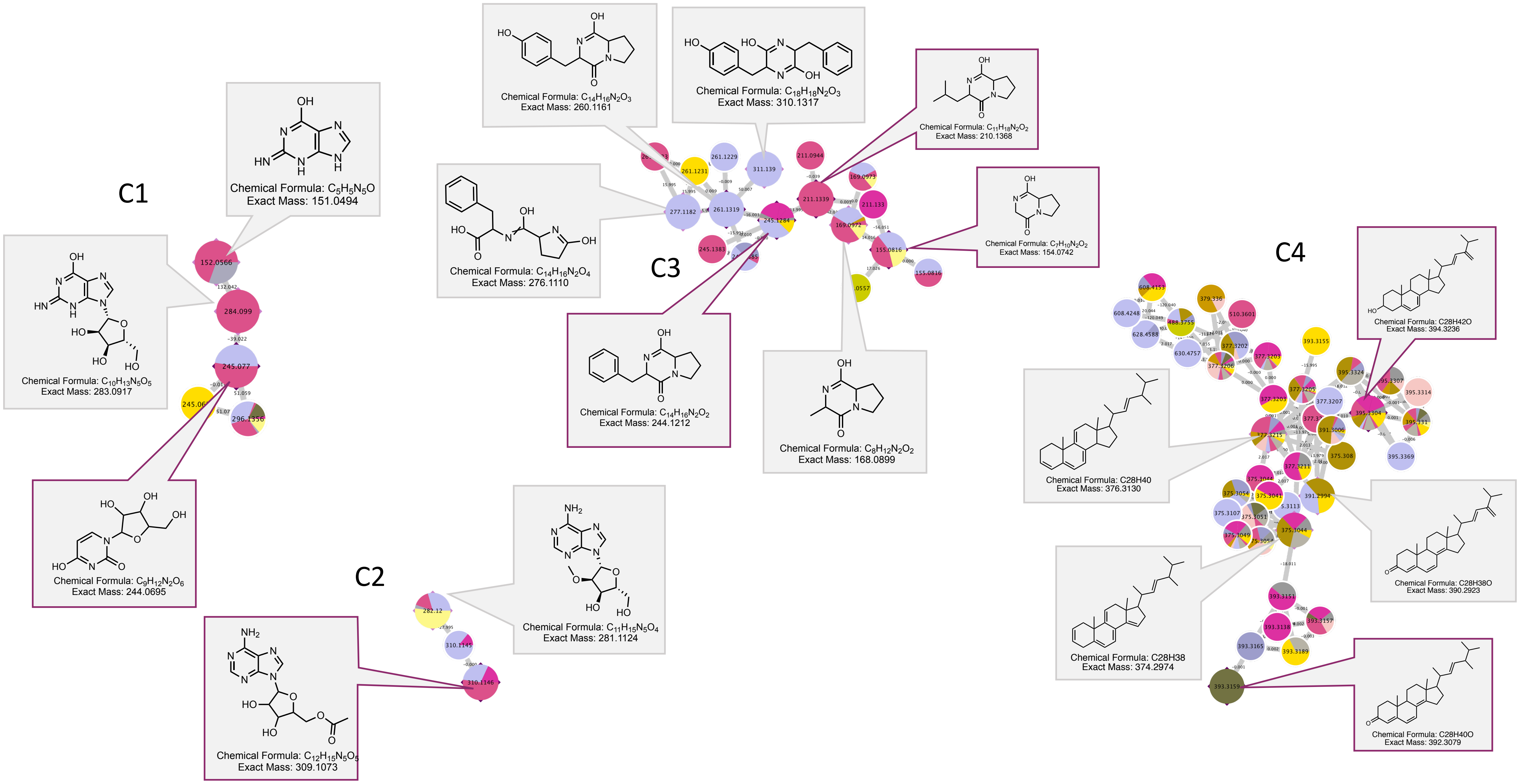

Figure S11. Purple box: features identified using standards and previously isolated molecules; grey box: propagation of annotation to neighbouring features

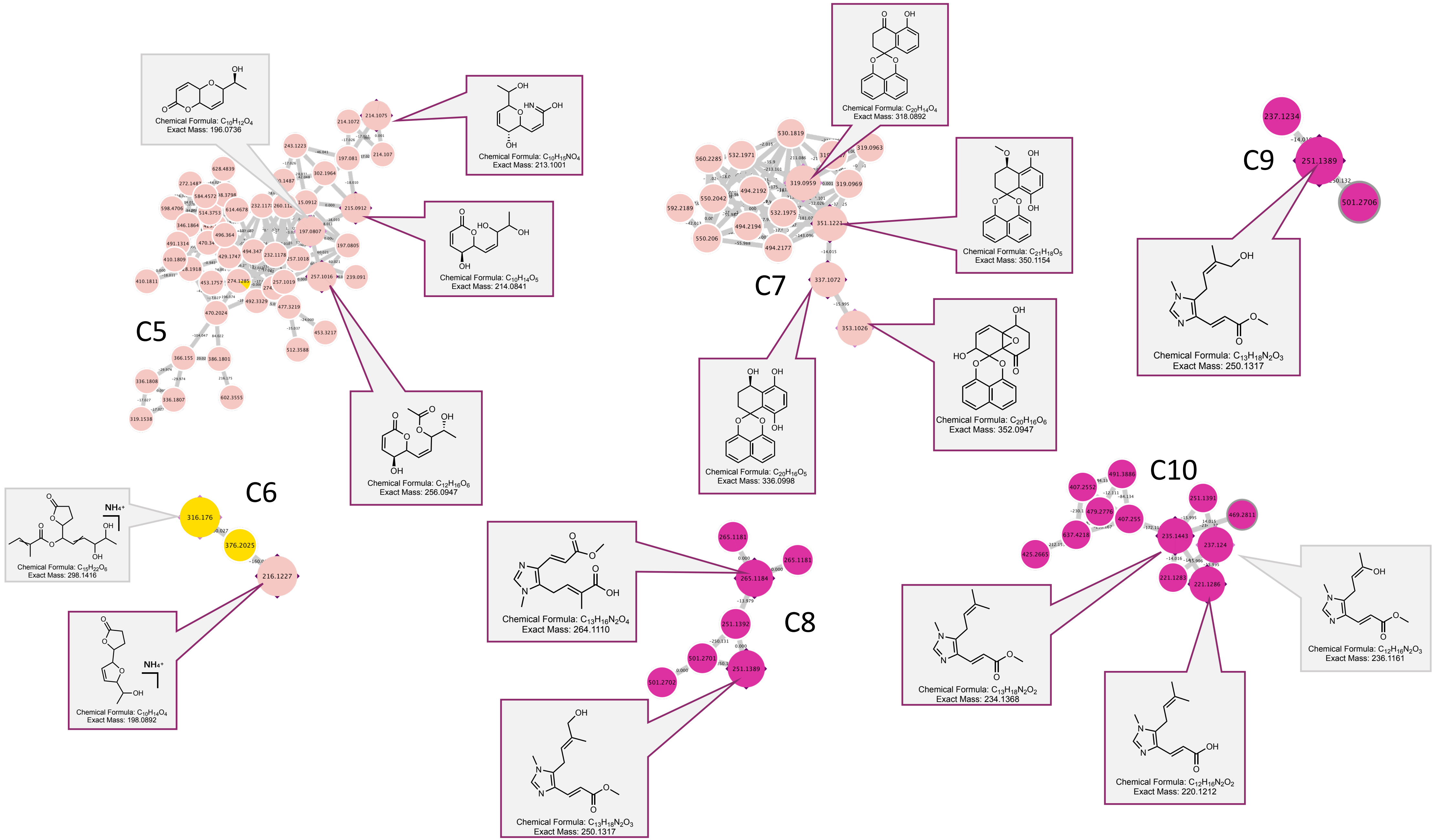

Figure S12. Purple box: features identified using standards and previously isolated molecules; grey box: propagation of annotation to neighbouring features.

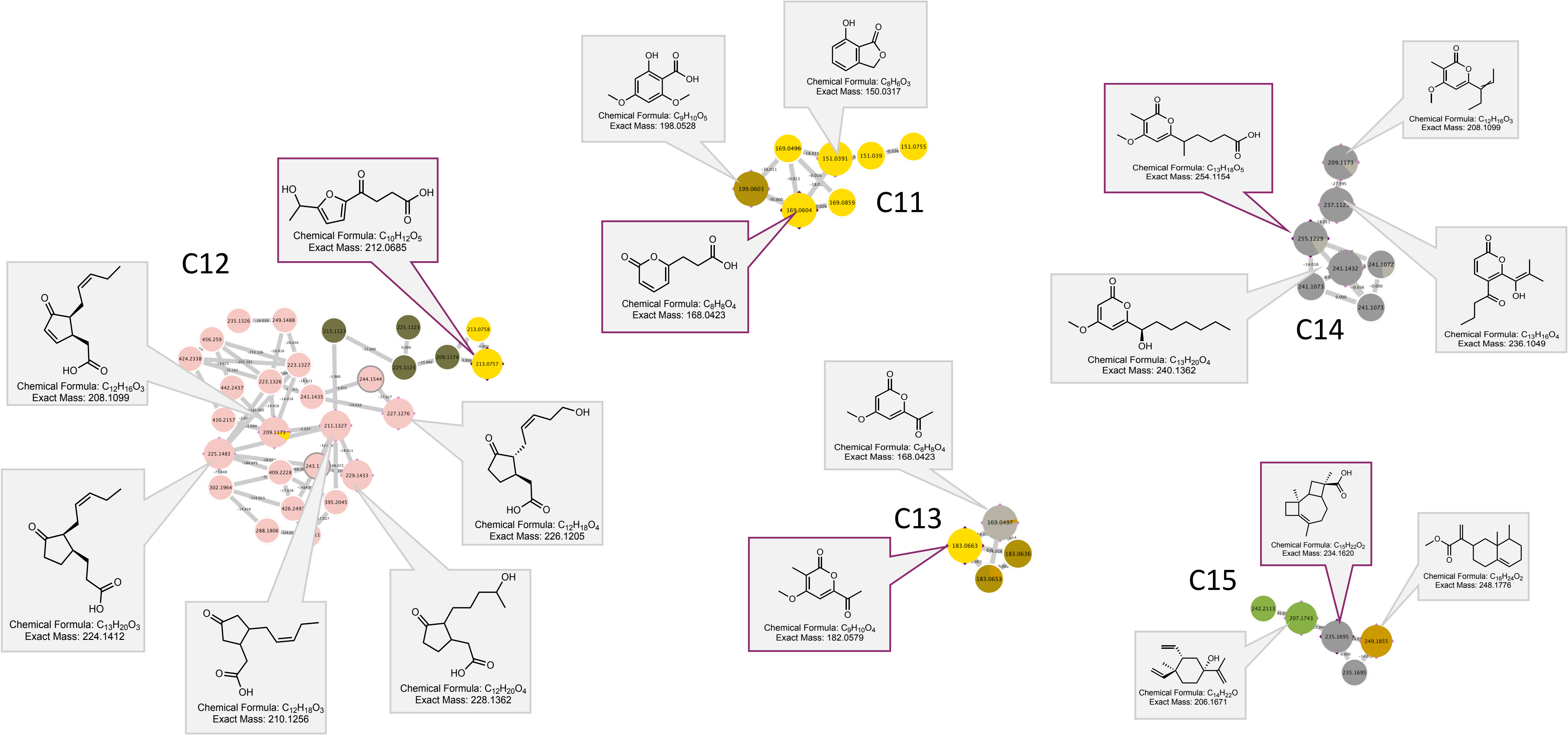

Figure S13. Purple box: features identified using standards and previously isolated molecules; grey box: propagation of annotation to neighbouring features

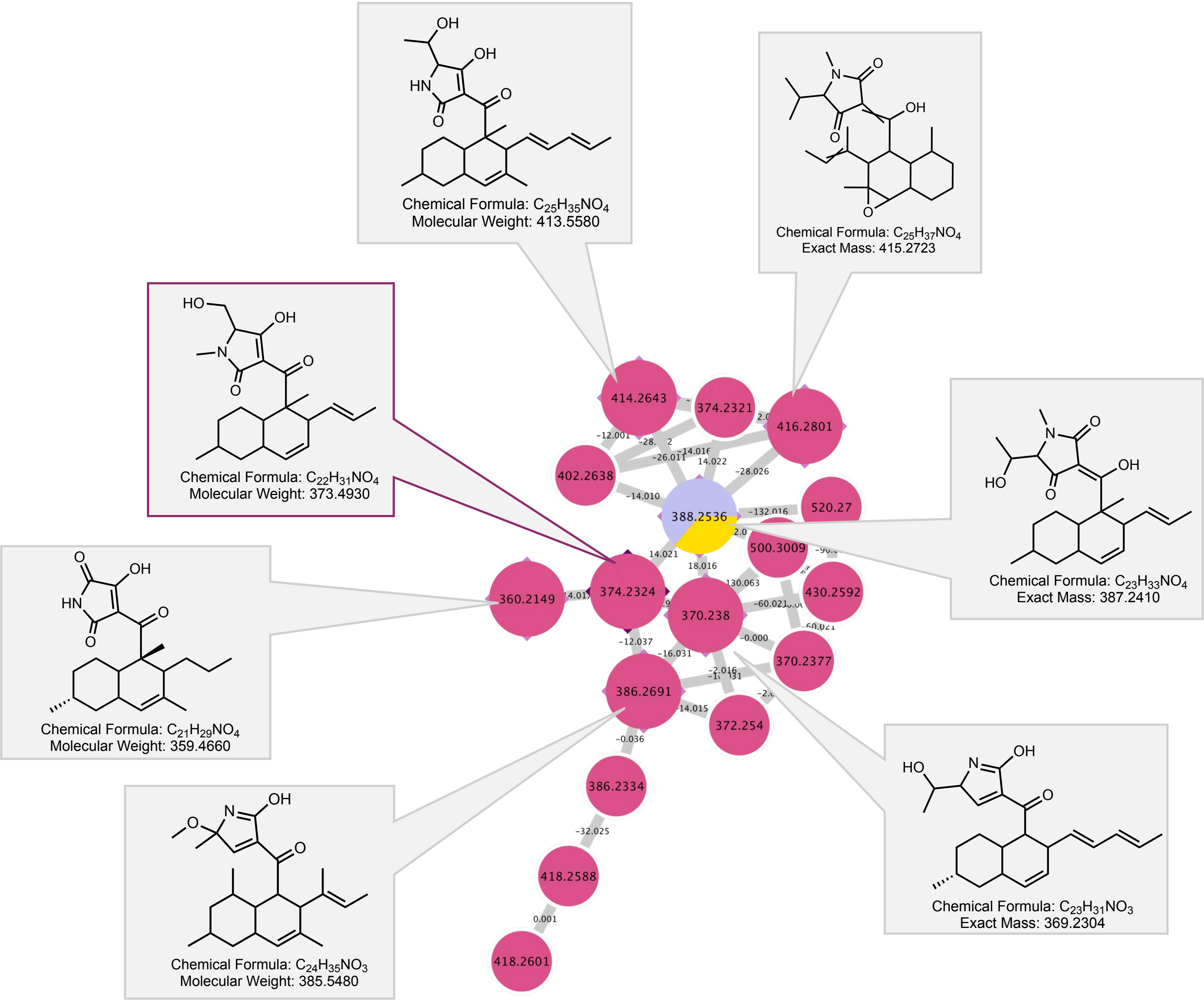

Figure S14. Cluster representing equisetins

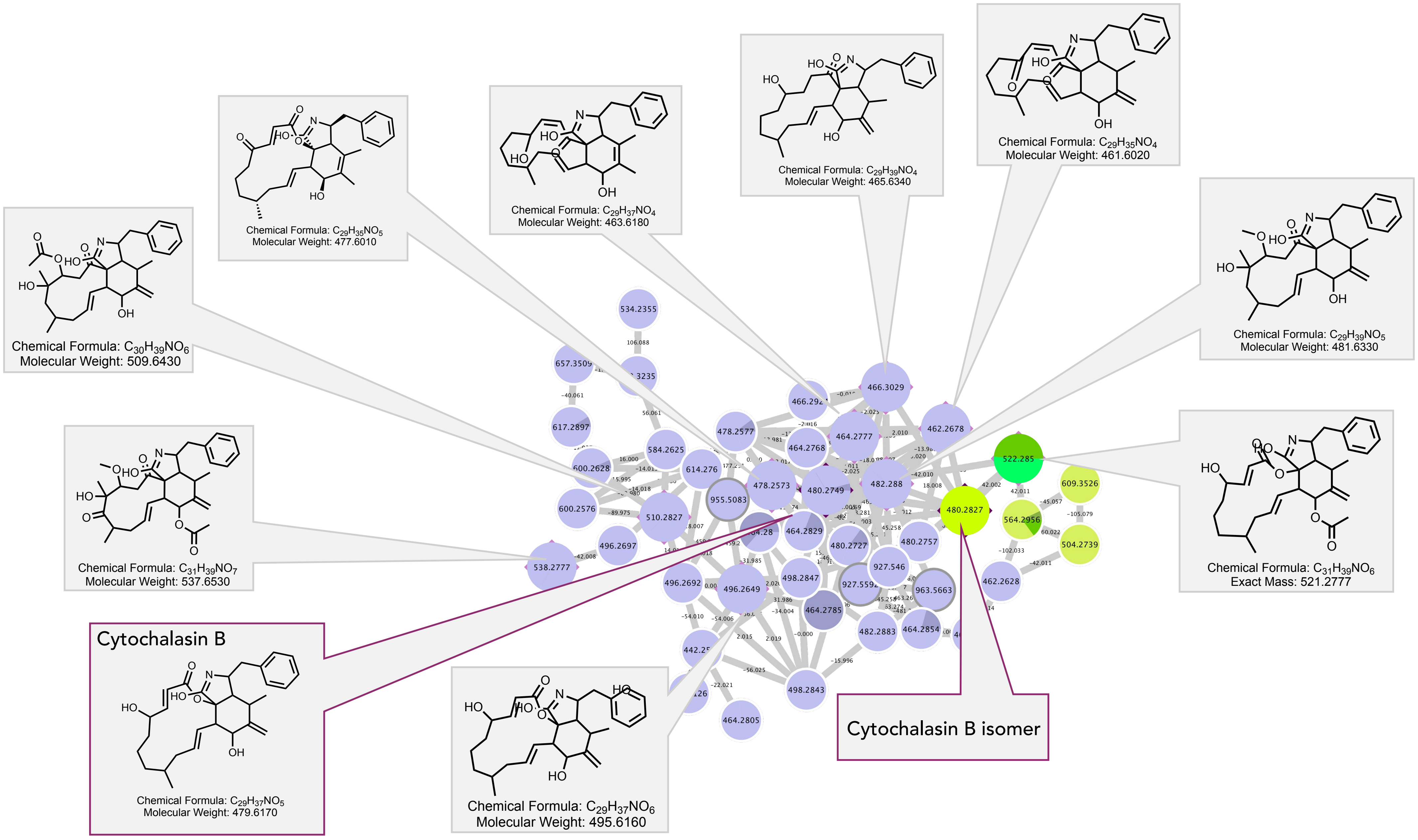

Figure S15. Purple box: features identified using standards and previously isolated molecules; grey box: propagation of annotation to neighbouring features

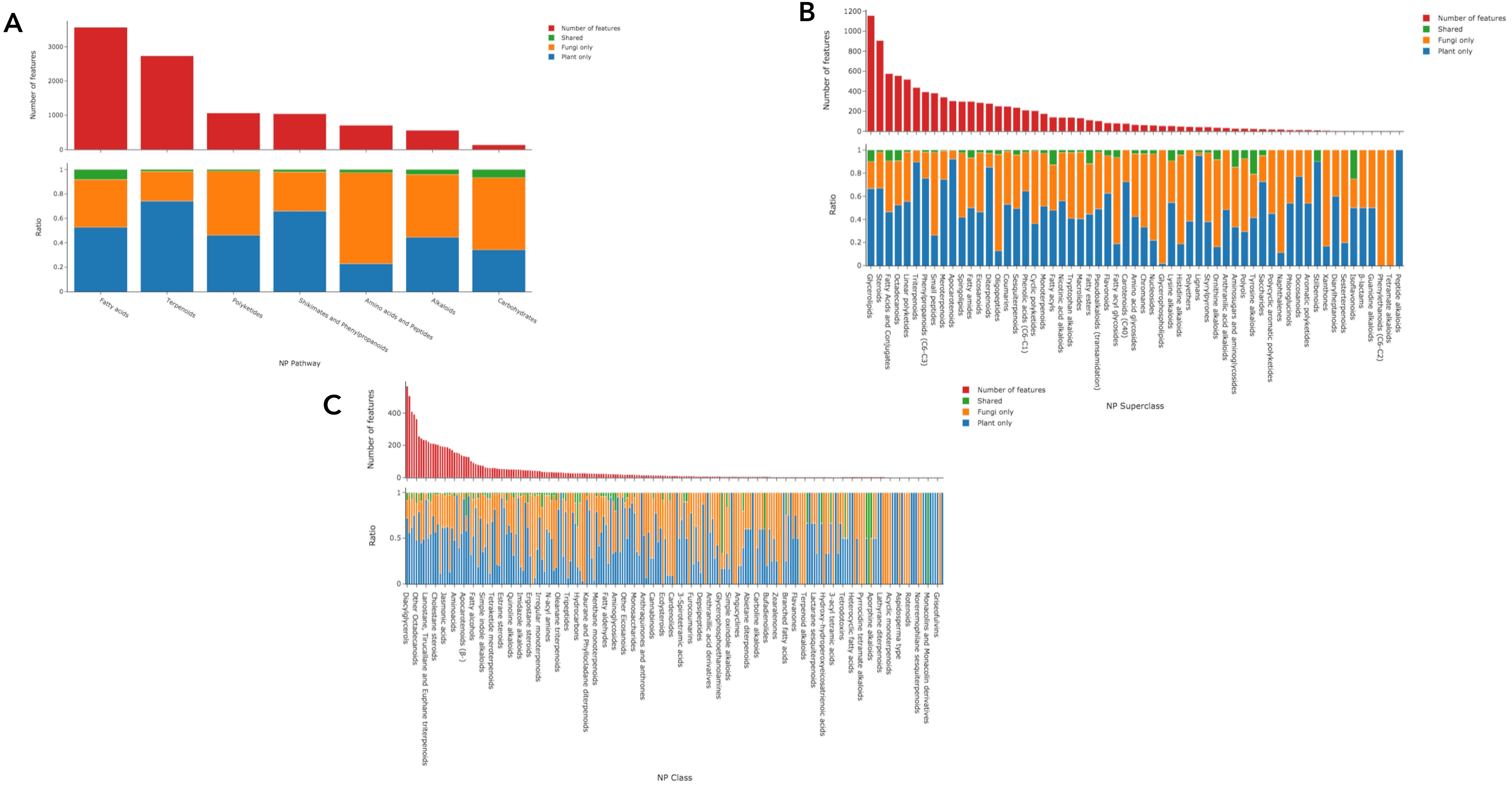

Figure S16. Number (red) and distribution of annotated features in the fungal (orange), plant (blue), or both (green) datasets, (A) at the pathway level, (B) at the superclass level, (C) at the class level (for full view, see Interactiveplots).

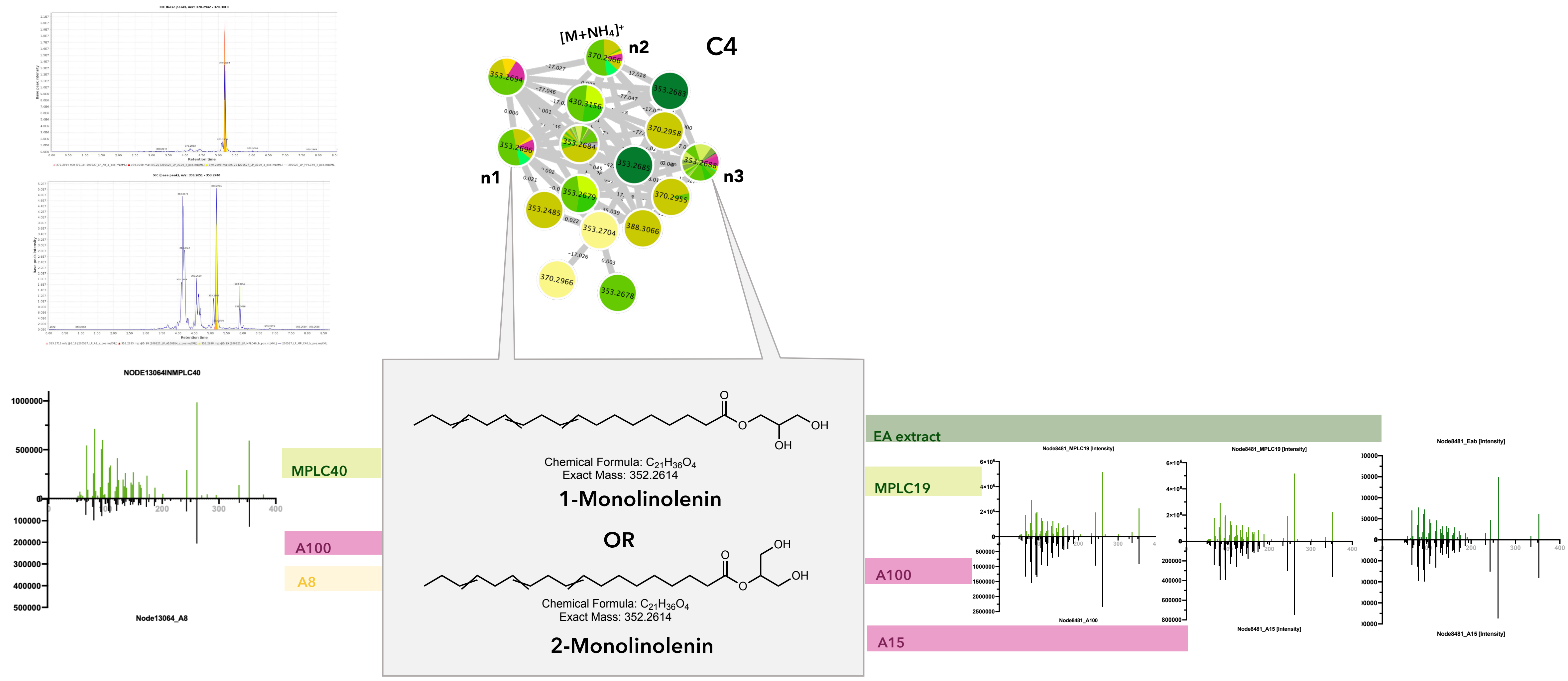

Figure S17. Cluster C4 representing a cluster of shared glycerolipids (MAGs).

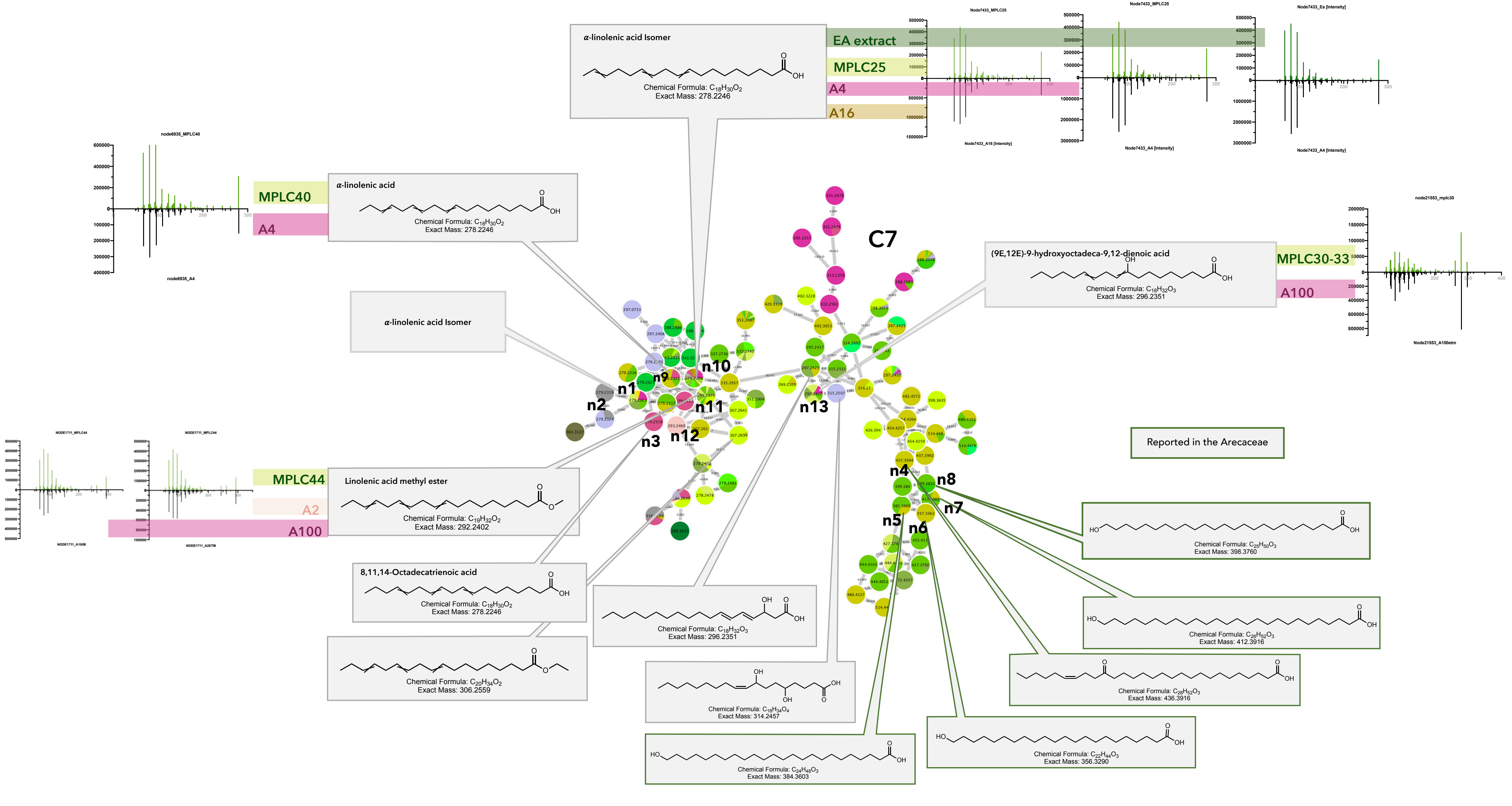

Figure S18. Cluster C7 representing a cluster of shared fatty acids conjugates.

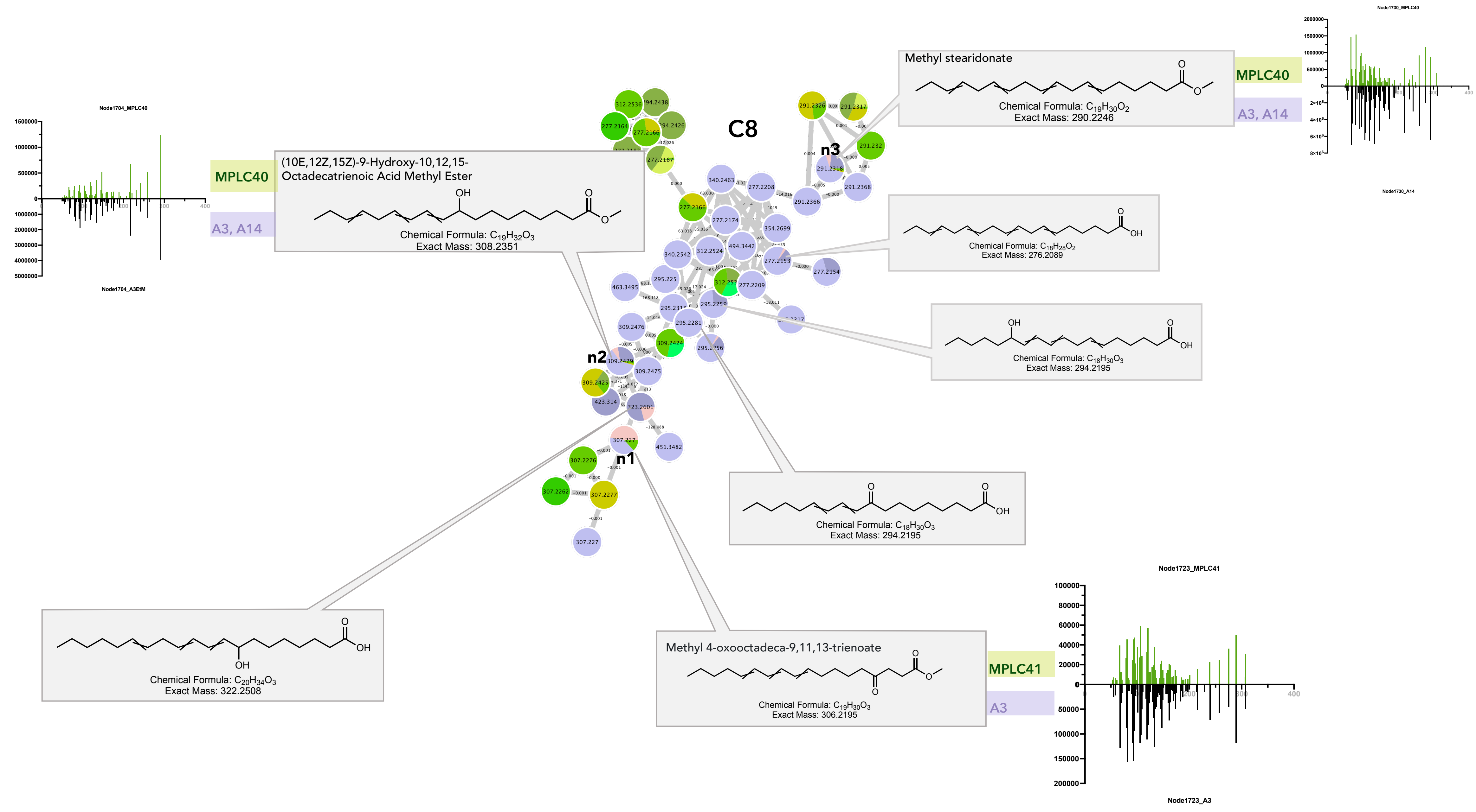

Figure S19. Cluster C8 representing a cluster of shared unsaturated fatty acids.

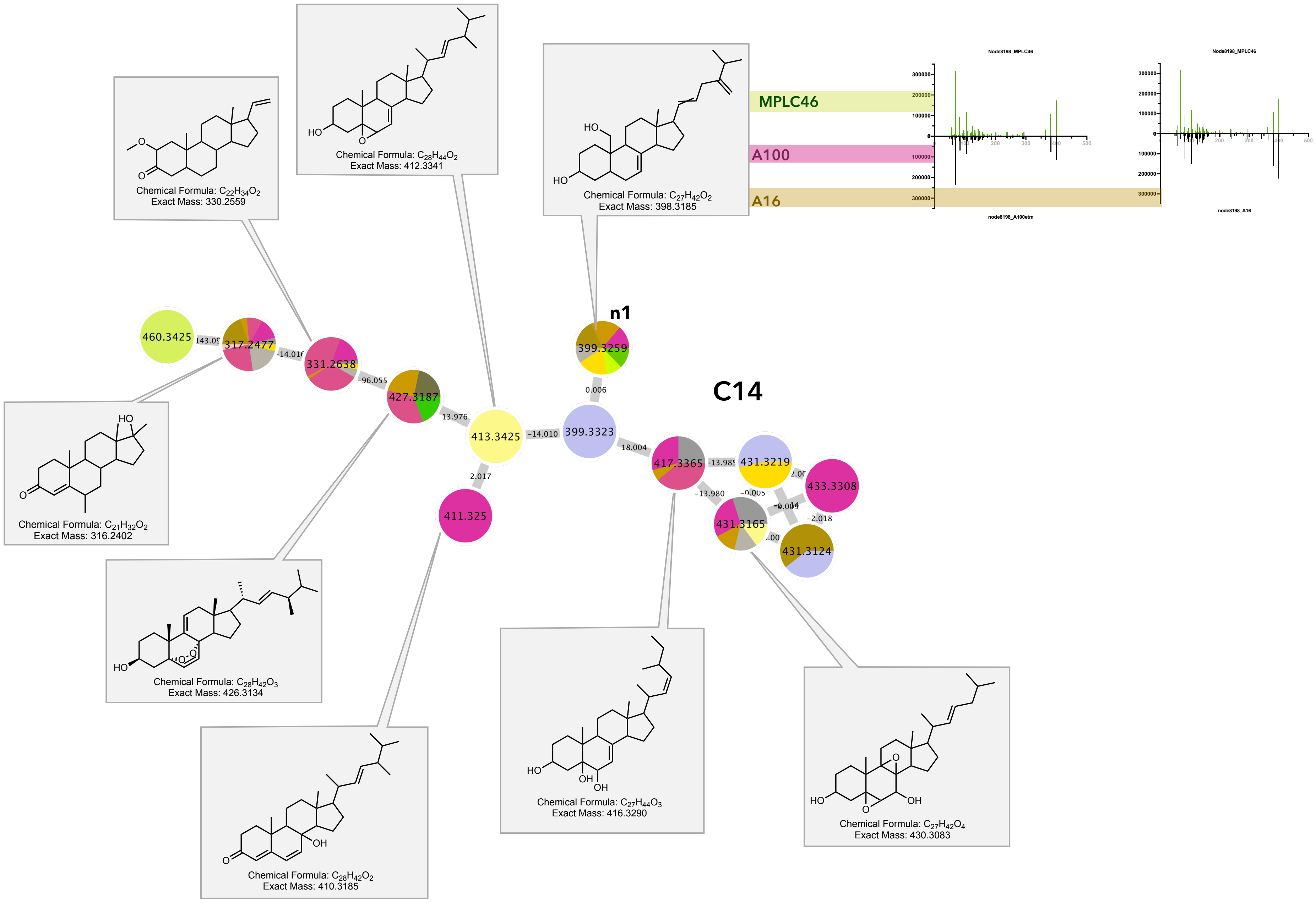

Figure S20 Cluster C14 representing a cluster of shared steroid derivatives.

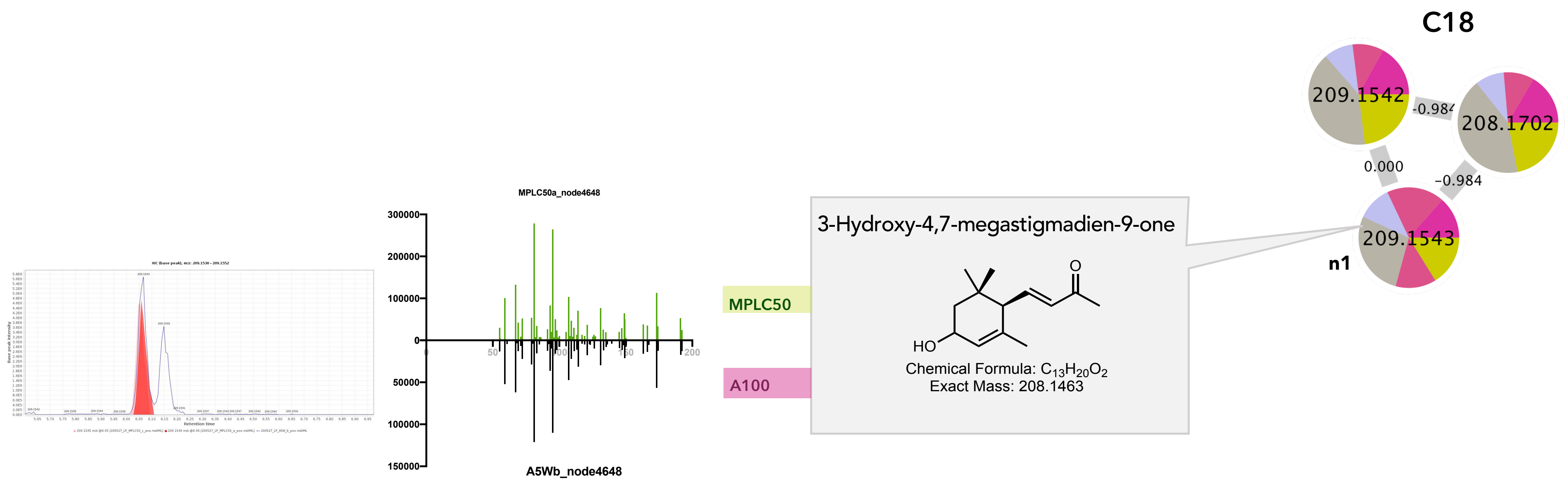

Figure S21 Cluster C18 representing a cluster of shared apocarotenoids.
